# Meta-analysis of elastic versus rigid fixation in the treatment of acute tibiofibular syndesmosis injury

**DOI:** 10.1186/s13643-023-02448-2

**Published:** 2024-02-02

**Authors:** Qin Wang, Shuan Liu, Zhemin Wang, Ao Li, Jinhui Ding

**Affiliations:** Tianyou Hospital affiliated to Wuhan University of Science and Technology, Wuhan, Hubei 430064 China

**Keywords:** Tibiofibular syndesmosis injury, Surgical fixation, Elastic fixation, Rigid fixation, Meta-analysis

## Abstract

**Objective:**

The objective of this study was to conduct a meta-analysis by synthesizing multiple literature sources to explore whether there are any differences between elastic fixation and rigid fixation in the treatment of acute tibiofibular syndesmosis injuries. The aim was to provide effective guidance for clinical treatment.

**Methods:**

We conducted a comprehensive search across seven databases, including both Chinese and English, to include all studies related to the treatment of acute tibiofibular syndesmosis injuries with elastic fixation and rigid fixation published between January 1, 2013, and November 15, 2022. Following the PRISMA guidelines, we rigorously screened, assessed, and extracted data from the included studies. The outcome measures included AOFAS scores at 3, 6, and 12 months postoperatively; tibiofibular clear space (TBCS) and tibiofibular overlap distance (TBOL) at the early postoperative and 12-month follow-up; intraoperative blood loss; operative time; time to full weight-bearing postoperatively; and postoperative complications. Meta-analysis was performed using Review Manager 5.4.

**Results:**

A total of 35 studies were included, comprising 16 randomized controlled trials and 19 retrospective cohort studies. The study population included 2120 cases, with 1044 cases in the elastic fixation group and 1076 cases in the rigid fixation group. The elastic fixation group had higher AOFAS scores at 3, 6, and 12 months postoperatively compared to the rigid fixation group. Although the elastic fixation group had a slightly larger TBCS than the rigid fixation group in the early postoperative period, the difference between the two groups became statistically insignificant at 12 months postoperatively. There was no statistically significant difference in TBOL between the two groups in the early postoperative period, but at 12 months, the elastic fixation group had a greater TBOL than the rigid fixation group. Additionally, the elastic fixation group had lower rates of postoperative local irritation, wound infection, and postoperative internal fixation loosening or rupture compared to the rigid fixation group. The rate of postoperative tibiofibular redislocation did not differ statistically between the two groups. The time to full weight-bearing was shorter in the elastic fixation group than in the rigid fixation group. Although the elastic fixation group had a slightly longer operative time, there was no statistically significant difference in intraoperative blood loss between the two groups.

**Conclusion:**

Compared to rigid fixation, elastic fixation in the treatment of acute tibiofibular syndesmosis injuries offers several advantages, including better postoperative ankle joint function recovery, more precise anatomical reduction of the syndesmosis postoperatively, a lower incidence of postoperative complications, and shorter time to full weight-bearing postoperatively. These findings provide robust guidance for clinical treatment.

**Supplementary Information:**

The online version contains supplementary material available at 10.1186/s13643-023-02448-2.

## Introduction

Ankle joints are composed of the distal tibia and fibula, the talus bone, and the surrounding ligaments [1]. As the largest weight-bearing hinge joint in the human body, the ankle joint plays a crucial role in both movement and weight transmission. Due to its complex structure and significant mechanical properties during motion, ankle fractures are among the most common types of bone injuries. It is estimated that the incidence of syndesmotic ligament rupture in all surgically treated ankle fractures ranges from 39 to 45% [2]. Consequently, stabilizing syndesmotic separation, reconstructing the ankle mortise, and restoring ankle joint anatomical congruency have become essential aspects of ankle fracture treatment.

For decades, the use of one or more screws passing through three or four layers of cortical bone for fixing syndesmotic separation after ankle fracture reduction has shown definite clinical efficacy and has become the gold standard for surgical treatment of syndesmotic injuries according to orthopedic association guidelines. However, the rigidity of screw fixation hinders the physiological movement of the fibula within the tibial groove, leading to drawbacks such as screw loosening, breakage, loss of syndesmotic reduction, and the potential need for secondary removal of internal fixation [3]. Therefore, to align with the physiological characteristics of the tibiofibular syndesmotic articulation, a more flexible suture-button plate system has been introduced. In theory, an elastic fixation system can address the various issues arising from the mismatch between screw rigidity and syndesmotic micromotion characteristics [4]. Nevertheless, in the practical clinical application, the question of which fixation technique can provide superior clinical outcomes remains a subject of debate.

Hence, this study conducts a comprehensive meta-analysis by synthesizing data from multiple articles, aiming to objectively compare the pros and cons of elastic fixation versus rigid fixation in the treatment of acute syndesmotic injuries from a macroscopic biomechanical perspective. The goal is to provide valuable insights for clinical practitioners and offer research directions for new methods and materials in the treatment of syndesmotic injuries in the future.

## Methods

### Search strategy

We conducted searches across seven databases, including PubMed, CNKI (China National Knowledge Infrastructure), Wanfang Data, VIP Database, Sinomed, Embase, and The Cochrane Library. We collected literature published between January 1, 2013, and November 15, 2022, related to the treatment of tibiofibular syndesmotic injuries using elastic fixation and rigid fixation. The search strategy employed a combination of subject terms and free-text keywords. The primary search terms included ankle syndesmosis, articulatio talocruralis, tibiofibular ankle syndesmosis, distal tibiofibular joint, Suture-Button, Endobutton, TightRope, dynamic fixation, Bone Screws, rigid fixing, rigid fastening, “下胫腓联合损伤,” “下胫腓联合韧带损伤,” “下胫腓损伤,” “弹性固定,” “动态固定,” “带袢钢板,” “缝合纽扣,” “刚性固定,” “静态固定,” and “螺钉.”

### Inclusion and exclusion criteria

Inclusion criteria were as follows: (1) inclusion of literature comparing elastic fixation and rigid fixation for the treatment of acute tibiofibular syndesmotic injuries, (2) inclusion of literature with clear radiological evidence or intraoperative confirmation using the Cotton or Hook test to diagnose tibiofibular syndesmotic injuries, (3) inclusion of literature involving patients aged between 18 and 60 years, and (4) inclusion of literature with patients who had normal pre-injury limb function, no prior history of ankle fractures or ligament injuries in the affected limb, and no documented repeated manual reductions.

Exclusion criteria were as follows: (1) exclusion of literature lacking evidence confirming the inclusion cases as acute tibiofibular syndesmotic injuries; (2) exclusion of literature with poorly designed experiments or inadequate data; (3) exclusion of duplicated publications, as well as reviews, systematic reviews, comments, animal experiments, model studies, and case reports; (4) exclusion of literature involving cases with comorbidities such as diabetes, metabolic syndrome, or severe osteoporosis that could impact the prognosis; (5) exclusion of literature involving open fractures, pathological fractures, or cases with severe vascular or nerve injuries; and (6) exclusion of literature with a follow-up period of less than 6 months or a loss to follow-up rate exceeding 20%.

### Quality assessment of included studies

All included literature was independently assessed by two investigators following a double-blind principle. Quality assessment of the retrieved literature was conducted using the Cochrane Risk Assessment Scale and the NOS Assessment Scale. For randomized controlled trials, the Cochrane Risk Assessment Scale was employed. Literature meeting three or more criteria on the scale was included. For retrospective cohort studies, the NOS Assessment Scale was utilized. Literature scoring six stars or higher on the scale was included.

### Data extraction and statistical analysis

Two independent reviewers thoroughly reviewed all included literature. They compiled a standardized Excel spreadsheet that included the following information: publication year, first author’s name, study design type, blinding method (if used), quantity and type of elastic fixation, type and method of rigid fixation, sample size, gender ratio, age, type of ankle joint fracture, follow-up duration, postoperative AOFAS score, TBCS, TBOL, surgical duration, postoperative complications, full weight-bearing time after surgery, and intraoperative blood loss.

This study utilized Revman Manager 5.4 provided by Cochrane Collaboration for analysis. When *I*^2^ < 50% and the *p*-value of the *Q*-test was ≥ 0.1, it was considered that there was no statistically significant heterogeneity among the included studies, and a fixed-effects model was applied for analysis. When *I*^2^ ≥ 50% or the *p*-value of the Q-test was < 0.1, it was deemed that there was statistically significant heterogeneity among the included studies. In such cases, sensitivity analysis or subgroup analysis was conducted to eliminate heterogeneity, and then, a fixed-effects model was used for analysis. For literature in which heterogeneity could not be eliminated, a random-effects model was employed for analysis.

## Results

### Literature search and selection

**Fig. 1:**
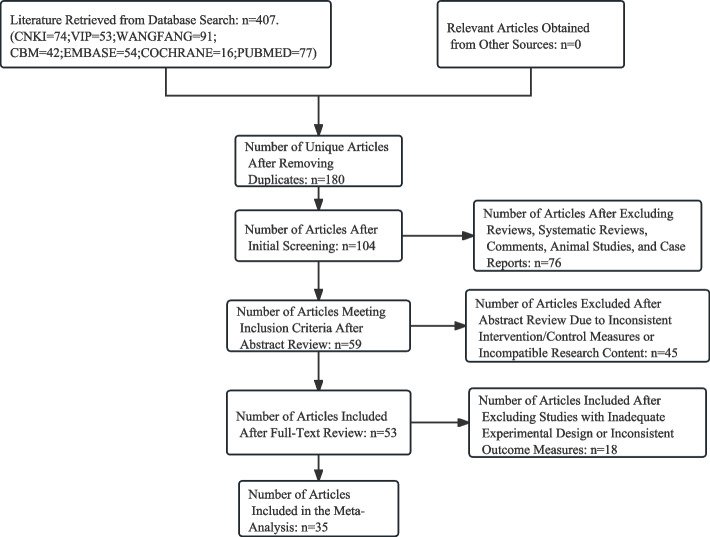
Study flow diagram

From seven databases, including PubMed, CNKI, VIP, Wanfang, Sinomed, Embase, and Cochrane, a total of 407 relevant articles on the subject were initially retrieved. Duplicate articles were removed, and exclusions were made for systematic reviews, meta-analyses, commentaries, animal experiments, and case reports. Further exclusions were based on inconsistent outcome measures, poorly designed experiments, mismatched research methods, unrelated study content, and discrepancies in intervention or control measures. Ultimately, 35 articles were selected for inclusion in the meta-analysis, as illustrated in Fig. 1.

### Characteristics of the eligible studies

Included were 35 articles in total. Among these, 16 were randomized controlled trials with Cochrane Risk Assessment Scores of 4 or higher. The remaining 19 were retrospective cohort studies with NOS quality assessment scores of 6 or higher. The types of elastic fixation devices used in the literature included TightRope, Endobutton, Nice knot (NICE), suture-button, and others. The rigid fixation group, on the other hand, utilized a single 3.5-mm or 4.5-mm cortical bone screw penetrating 3 or 4 layers of cortical bone for the treatment of acute tibiofibular syndesmotic injuries. (See Supplementary Material 1 for a summary table of basic information on the literature, risk assessment charts, risk assessment table, and NOS quality assessment table).

## Conclusion

Regarding functional measures assessed by AOFAS scores, the AOFAS scores at 3, 6, and 12 months post-surgery were all significantly higher in the elastic fixation group compared to the rigid fixation group in the treatment of acute tibiofibular syndesmotic injuries (*P* < 0.05). Thus, when using the AOFAS scoring system as the clinical efficacy measure for treating acute tibiofibular syndesmotic injuries, elastic fixation devices exhibited superior clinical outcomes at 3, 6, and 12 months postoperatively compared to rigid fixation devices. Notably, the AOFAS scores for elastic fixation, particularly at 3 months postoperatively, demonstrated a significant advantage over rigid fixation, suggesting that elastic fixation excels in early postoperative ankle function recovery.

In terms of radiological indicators, TBCS and TBOL, at the early postoperative stage, the rigid fixation group exhibited a smaller tibiofibular clear space compared to the elastic fixation group (*P* < 0.05). However, at 12 months post-surgery, no statistically significant difference in tibiofibular clear space was observed between the two groups (*P* > 0.05), indicating that elastic fixation can provide conditions for tibiofibular ligament recovery similar to rigid fixation. The tibiofibular overlap showed no statistically significant difference between the two groups at the early postoperative stage (*P* > 0.05), but at 12 months post-surgery, the elastic fixation group exhibited greater tibiofibular overlap than the rigid fixation group (*P* < 0.05), suggesting that elastic fixation may lead to better anatomical realignment of the fibula within the tibial incision.

In the analysis of postoperative complications, including local irritation and wound infection, internal fixation loosening or rupture, and tibiofibular redislocation, elastic fixation demonstrated lower overall postoperative complication rates, lower rates of local irritation and wound infection, and lower rates of internal fixation loosening or rupture compared to rigid fixation (*P* < 0.05). However, there was no statistically significant difference in the rate of tibiofibular redislocation between the two groups (*P* > 0.05).

Elastic fixation also resulted in a shorter time to full weight-bearing postoperatively compared to rigid fixation (*P* < 0.05), albeit with slightly longer surgical times (*P* < 0.05). The difference in intraoperative blood loss between the two groups was not statistically significant (*P* > 0.05).

In summary, compared to rigid fixation, elastic fixation has advantages in terms of better postoperative ankle joint function recovery, lower postoperative complication rates, and shorter time to full weight-bearing in the treatment of acute tibiofibular syndesmotic injuries, as shown in Table 1. (See Supplementary Material 2 for meta-analysis forest plots, funnel plots, and the analysis process).

**Table 1:** Incorporating the Meta-analysis results of the included research literature

**Outcome Measures**	**Included RCTs**	**Included RCSs**	**Elastic Fixation** **Cases**	**Rigid Fixatio** **n Cases**	**Heteroge neity Test** **(I'%)**	**Test Model**	**Combined Statistic**	**Combined Statistic Value (95%*Cl)***	***P-value***
3-month postoperative AOFAS score [5–12]	3	5	181	185	40%	Fixed Effects	MD	9.05 [6.90, 11.19]	< 0.05
6-month postoperative AOFAS score [5, 6, 8, 10–20]	7	5	424	436	28%	Fixed Effects	MD	1.59 [0.51, 2.68]	< 0.05
12-month postoperative AOFAS score [5–8, 10–14, 16, 17, 19–25]	11	7	495	517	0%	Fixed Effects	MD	2.00 [1.28, 2.72]	< 0.05
Early postoperative TBCS [9, 10, 17, 25–32]	2	9	292	313	28%	Fixed Effects	MD	0.19 [0.12, 0.25]	< 0.05
12-month postoperative TBCS[1, 6, 10, 16, 17, 21, 31–35]	3	8	267	284	3%	Fixed Effects	MD	-0.06 [—0.14, 0.02]	0.12
Early postoperative TBOL [9, 10, 17, 25–32]	2	9	292	313	38%	Fixed Effects	MD	0.06 [-0.04, 0.15]	0.23
12-month Postoperative TBOL [1, 6, 10, 16, 17, 21, 31–35]	3	8	279	310	25%	Fixed Effects	MD	0.13 [0.03, 0.24]	< 0.05
Postoperative local irritation and wound infection rate [9–11, 13–15, 17, 18, 28, 30, 31, 36, 37]	6	7	490	465	9%	Fixed Effects	RR	0.54 [0.32, 0.93]	< 0.05
Postoperative internal fixation loosening or rapture rate [9–11, 13, 15, 17, 18, 28, 30, 31, 36, 37]	6	7	490	465	0%	Fixed Effects	RR	0.26 [0.12, 0.55]	< 0.05
Postoperative tibiofibular redislocation rate [9–11, 13, 15, 17, 18, 28, 30, 31, 36, 37]	3	3	490	465	0%	Fixed Effects	RR	0.74 [0.28, 1.91]	0.53
Postoperative full weight-bearing time [9, 10, 13, 15, 17, 18, 28–30, 34, 36]	5	6	412	411	94%	Random Effects	MD	-1.69 [—2.39,—0.92]	< 0.05
Intraoperative blood loss [9, 12, 15, 18, 24, 26–28, 30, 33–35, 38]	4	9	407	421	0%	Fixed Effects	MD	-0.73 [—1.76, 0.31]	0.17
Surgical time [12, 15, 18, 24, 26–28, 30, 33–35, 38]	6	6	346	366	96%	Random Effects	MD	9.24 [2.91, 15.57]	< 0.05

## Discussion

The ankle joint, as the body’s largest “mortise” joint, forms the “mortise” by connecting the distal tibia and fibula through the syndesmosis, allowing stable motion of the talus within. The syndesmosis mainly consists of the anterior inferior tibiofibular ligament (AITFL), interosseous ligament, and posterior inferior tibiofibular ligament (PITFL) [39]. The AITFL primarily resists fibular external rotation and posterior translation, while the PITFL mainly resists fibular internal rotation [40]. Lauge Hansen’s pronation-external rotation (Weber B) and pronation-abduction-external rotation (Weber C) injuries are the most common injuries that affect syndesmosis [2]. Once the precise alignment of the distal tibia and fibula is disrupted, the stability of the ankle joint is compromised. For every 1-mm increase in the tibiofibular diastasis, the contact area of the tibiotalar joint decreases by 42% [41], leading to increased cartilage wear and the potential development of post-traumatic osteoarthritis. Therefore, surgical intervention is crucial for the treatment of syndesmotic injuries, restoring stability, maintaining ankle joint function, and preventing the progression of joint arthritis.

While syndesmotic injuries are relatively common, there is still controversy regarding the optimal fixation method for unstable syndesmosis. Numerous cadaveric and biomechanical studies have shown that both elastic and rigid fixation can provide sufficient resistance to separation stress [42]. However, our study revealed a higher incidence of loosening or fracture in rigid fixation postoperatively compared to elastic fixation. Interestingly, there was no statistically significant difference in the postoperative redislocation rate between elastic and rigid fixation. This implies that elastic fixation may not offer separation resistance similar to rigid fixation, or the mechanical characteristics of micro-motion in elastic fixation may not provide a stable environment for syndesmotic repair. Further exploration of these findings may be necessary.

Improved anatomical reduction may lead to better results. When a difference of 1.5 to 2 mm in syndesmotic width between bilateral ankles is used as the threshold for malreduction, the malreduction rate for screw fixation ranges from 16 to 52% [43, 44]. Elastic fixation, due to its allowance of physiological motion between the fibula and tibia, may result in better anatomical reduction under stress. Some studies have shown that after 1 year of suture-button fixation, ligament consistency improved compared to preoperative and postoperative computed tomography (CT) scans [45]. Other studies suggest that both screw and suture-button fixation increase syndesmotic gap volume compared to the contralateral limb, but this increase is statistically significant only in elastic fixation [44]. Therefore, this study found that early postoperative tibiofibular diastasis was smaller in the rigid fixation group compared to the elastic fixation group, with no significant difference in tibiofibular overlap distance between the two groups. However, at 12 months postoperatively, the rigid fixation group had greater tibiofibular overlap distance than the elastic fixation group, with no significant difference in tibiofibular diastasis. This may be because elastic fixation can provide conditions for syndesmotic repair similar to rigid fixation and more accurate anatomical reduction.

Screw loosening, breakage, and the possibility of secondary removal surgeries are important factors affecting the clinical outcomes of rigid fixation. Due to the rigid properties of screws conflicting with the micromotion characteristics of the syndesmosis, rigid fixation devices may lead to loss of ankle joint mobility and may cause screw loosening and breakage under lower limb loading. According to AO principles, selective routine screw removal is performed 6–8 weeks after rigid fixation to restore physiological micromotion of the syndesmosis, relieve restrictions on ankle joint movement, and prevent discomfort caused by screw loosening and breakage [46]. However, secondary implant removal surgery carries risks such as infection, increased costs, and extended recovery time [4]. While some experts argue that screw removal offers no clear benefit [47], consensus has not been reached, and routine removal of tibiofibular screws remains a common practice clinically. Elastic fixation theoretically does not require implant removal, although the stimulus from implants (usually suture knots) can lead to a 6% removal rate of implants [43], but with the application of knotless elastic fixation devices, the removal rate of elastic fixation devices may be lower. Therefore, this study believes that, compared to rigid fixation, elastic fixation carries no risk of loosening and breakage and has a much lower rate of secondary implant removal.

However, elastic fixation also has its drawbacks. The surgical technique for elastic fixation of syndesmotic injuries is considered more complex than that for rigid fixation, requiring more surgical time and greater surgical experience to complete. This study also found that the surgical time for elastic fixation devices was slightly longer than that for rigid fixation devices, but rarely exceeded 90 min, which means there was no significant difference in intraoperative bleeding between elastic and rigid fixation. However, longer surgical time also implies a higher risk of infection. Although this study did not find a higher infection rate after rigid fixation compared to elastic fixation, another meta-analysis found that elastic fixation may have a higher rate of deep infection [OR = 1.40, 95% CI (0.40 ~ 4.85), *p* = 0.60] [48]. The braided sutures in elastic fixation devices may provide a favorable environment for the development of cross-joint fixation tunnel infections, which are closely related to bone resorption, tibiofibular bone drilling, and fixation device displacement [49]. Additionally, due to the flexibility of elastic fixation, it may not provide sufficient stability to resist fibular external rotation and posterior translation in severe ankle fractures or in patients with a high BMI, potentially compromising the conditions for syndesmotic ligament stability.

Cost considerations also significantly influence the choice of surgical method. The material cost of elastic fixation devices is much higher than that of rigid fixation devices. However, considering the combined costs of secondary removal of internal fixation, rehabilitation, and time off work, the total cost of elastic fixation device treatment for syndesmotic separation may be lower. Weber [50] and colleagues found that costs were equal at removal rates of 18 to 53% of tibiofibular screws. When 100% of tibiofibular screws were removed, elastic fixation was more cost-effective.

For individuals with higher demands for physical activity, elastic fixation devices may be a better choice because they do not pose a risk of implant breakage, do not restrict ankle joint mobility, and allow for earlier lower limb weight-bearing training, aiding in a faster return to pre-injury levels of physical activity. Colcuc et al. [51] found that the use of knotless TightRope fixation systems for syndesmotic injuries allowed patients to achieve faster rates and performance levels in both leisure and competitive sports.

In conclusion, compared to rigid fixation systems, elastic fixation systems may offer advantages such as better anatomical reduction, fewer complications, lower implant removal rates, lower overall costs, and improved physical performance. However, However, considering the drawbacks associated with existing elastic fixation methods like TightRope, Endobutton, NICE, and suture-button, there is a need for research into a more robust, cost-effective, and user-friendly elastic material for the treatment of acute syndesmotic injuries.

## Limitation

This meta-analysis aimed to investigate the clinical efficacy of elastic fixation compared to rigid fixation in the treatment of syndesmotic injuries, using AOFAS scores at 3, 6, and 12 months postoperatively as the primary outcome measures. Although the conclusions demonstrate that AOFAS scores favor elastic fixation at these time points over rigid fixation, it is important to note that the minimum clinically significant difference has not been definitively established. Therefore, there remains some controversy regarding the direct assessment of the clinical effectiveness difference between these two fixation methods using AOFAS scores. Furthermore, due to limitations in the included literature, this study only analyzed two radiographic assessment parameters, TBCS and TBOL based on X-ray images, which may not be sufficient to accurately evaluate the degree of syndesmotic reduction.

Since this study involves a comparison of different surgical approaches, there is a scarcity of double-blind randomized controlled trials among the included literature. Additionally, many of the studies are retrospective analyses, which could potentially impact the quality of evidence in this meta-analysis. Moreover, due to language limitations, this research only includes Chinese and English literature, possibly omitting high-quality studies in other languages, thus introducing a potential language bias.

### Supplementary Information

**Additional file 1:** **Supplementary Material 1.** Summary table of basic information on the literature, risk assessment charts, risk assessment table, and NOS quality assessment table.

**Additional file 2:** **Supplementary Material 2.** Meta-analysis forest plots, funnel plots, and the analysis process.

## Data Availability

The datasets used and/or analyzed during the current study are available from the corresponding author upon reasonable request.
