# Supplementary material for: Meta-analysis of elastic versus rigid fixation in the treatment of acute tibiofibular syndesmosis injury
Source: Syst Rev. 2024 Feb 2;13:51. doi: 10.1186/s13643-023-02448-2 (PMC10835897; doi:10.1186/s13643-023-02448-2)
Supplement: Supplementary file 1 — Additional file 1: Supplementary Material 1. Summary table of basic information on the literature, risk assessment charts, risk assessment table, and NOS quality assessment table. [file 13643_2023_2448_MOESM1_ESM.docx]

Among the final selection of 35 articles, 16 were categorized as randomized controlled trials (RCTs), while 19 were retrospective cohort studies. The elastic fixation devices used in the literature included TightRope,Endobutton, Nice Intramedullary Compression Expander (NICE), Suture-Button, and others. Notably, Endobutton was the most frequently employed elastic fixation device across the included studies. On the other hand, the rigid fixation groups consistently employed a single 3.5 mm or 4.5 mm cortical bone screw passing through three or four cortical layers for the treatment of acute tibiofibular syndesmotic injuries. The foundational characteristics of the included studies are illustrated in Table 1.

**Table 1 Basic information table**

#### Quality assessment of the eligible studies

The quality assessment of the included 16 randomized controlled trials (RCTs) was conducted using the Cochrane Risk of Bias Assessment Tool. All 16 articles achieved a score of 4 or higher, with 13 articles scoring 5 or above. One article (Laflamme 2015) scored 6 or above, as illustrated in Figures 2 and 3. Additionally, the quality analysis of the 19 included retrospective cohort studies was performed using the Newcastle-Ottawa Scale (NOS). All 19 articles scored 6 stars or above, with 10 articles achieving 7 stars or above, and 3 articles achieving 8 stars or above, as demonstrated in Table 2.

**
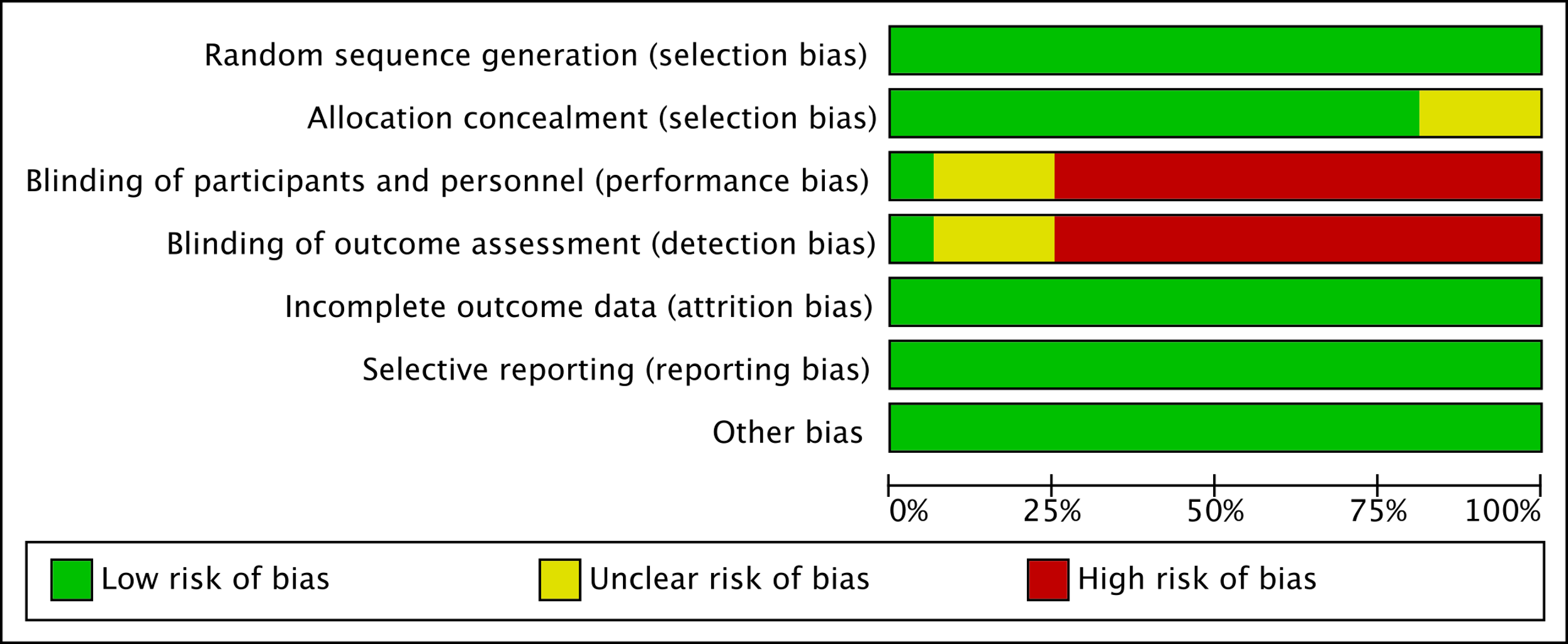
**

**Figure 2 Risk of bias graph**


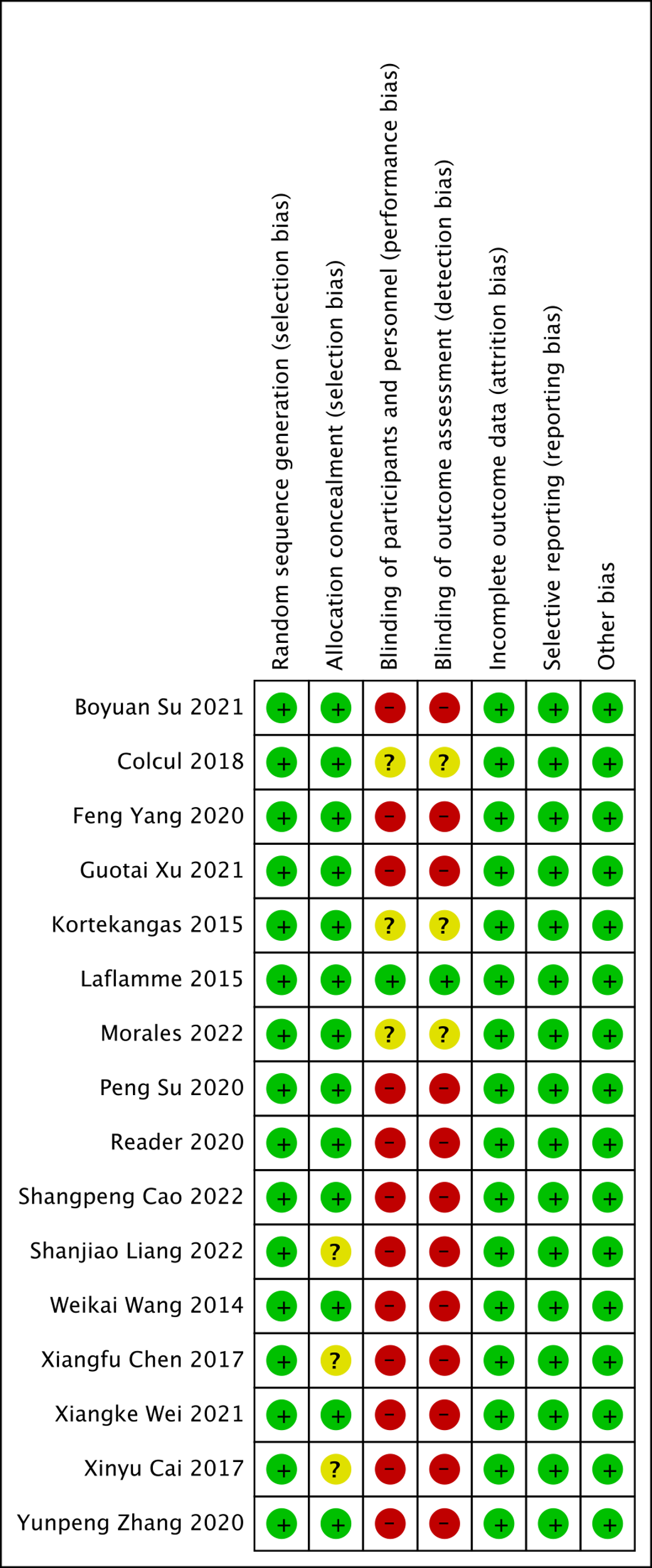


**Figure 3 Risk of bias summary**

**Table 2 Quality assessment of NOS**
