# Supplementary material for: Meta-analysis of elastic versus rigid fixation in the treatment of acute tibiofibular syndesmosis injury
Source: Syst Rev. 2024 Feb 2;13:51. doi: 10.1186/s13643-023-02448-2 (PMC10835897; doi:10.1186/s13643-023-02448-2)
Supplement: Supplementary file 2 — Additional file 2: Supplementary Material 2. Meta-analysis forest plots, funnel plots, and the analysis process. [file 13643_2023_2448_MOESM2_ESM.docx]

#### Meta- analysis outcome

AOFAS scores at 3, 6, and 12 months postoperatively.

1. AOFAS Scores at 3 Months Postoperatively

Eight articles^[5-12]^ were included for the analysis of AOFAS scores at 3 months postoperatively. Subgroup analyses were performed based on the study types, classifying the included studies into randomized controlled trials (RCTs) and retrospective cohort studies. Heterogeneity testing indicated a low overall heterogeneity among the 8 articles (I²=40%＜50%, and Q test's P=0.11＞0.1). Within the RCT subgroup, there was no heterogeneity observed (I²=0%, and Q test's P=0.42＞0.1). Similarly, within the retrospective cohort subgroup, there was no heterogeneity observed (I²=0%, and Q test's P=0.52＞0.1). While the overall heterogeneity across all 8 articles was weak (I²=40%, and Q test's P=0.11＞0.1), the heterogeneity between the two subgroups was high (I²=85.2%＞50%, and Q test's P=0.009＜0.1).

Fixed-effects meta-analysis was conducted, as shown in Figure 4. The combined mean difference of the AOFAS scores from all 8 articles was 9.05 and statistically significant (Z=7.88, P＜0.05). Among them, the combination of results from 3 RCTs showed a mean difference of 11.6 and was also statistically significant (Z=8.27, P＜0.05), indicating that the AOFAS scores at 3 months postoperatively were significantly higher in the elastic fixation group compared to the rigid fixation group. Furthermore, combining results from 5 retrospective cohort studies showed a mean difference of 5.89 and was statistically significant (Z=7.88, P＜0.05), indicating a similar trend of higher AOFAS scores in the elastic fixation group at 3 months postoperatively. Both the overall analysis and the subgroup analyses consistently demonstrated significantly higher AOFAS scores in the elastic fixation group at 3 months postoperatively compared to the rigid fixation group.

Publication bias was examined through the construction of a funnel plot, as depicted in Figure 5. The funnel plot from this study appeared symmetrical, indicating the absence of publication bias in the included literature.


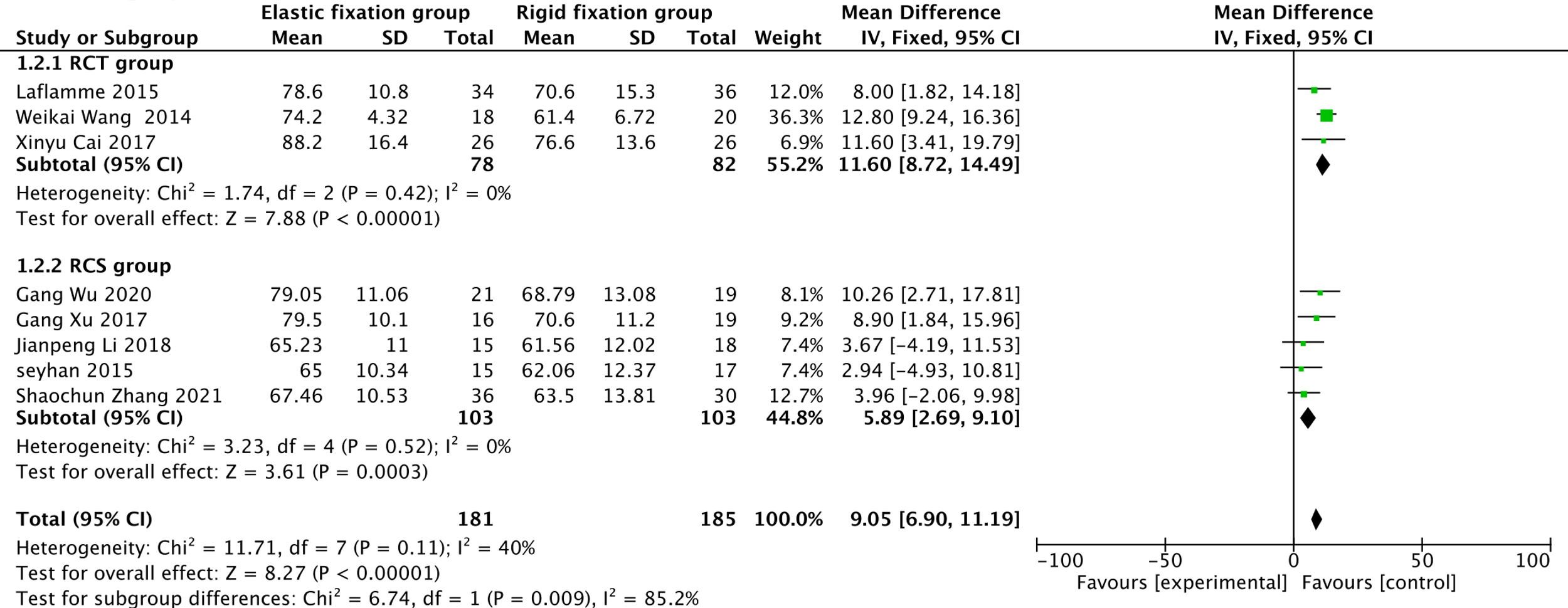


**Figure 4 AOFAS score forest plot 3 months after operation**


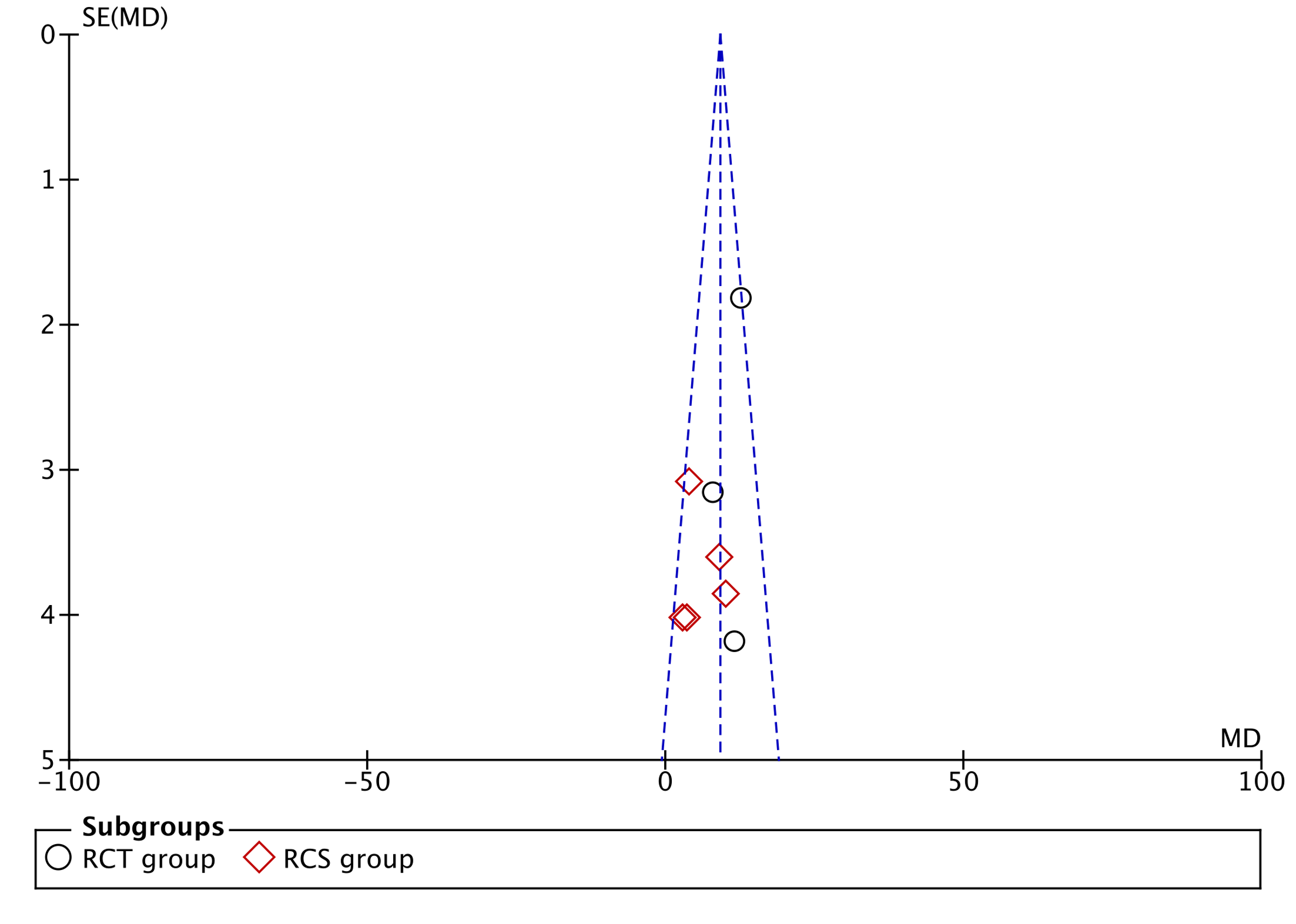


**Figure 5 AOFAS score funnel plot 3 months after operation**

2. AOFAS Scores at 6 Months Postoperatively

A total of 14 articles ^[5, 6, 8, 10-20]^ were included in the analysis of AOFAS scores at 6 months postoperatively. The included literature was divided into subgroups based on the study types: randomized controlled trials (RCTs) and retrospective cohort studies. The heterogeneity test revealed that the 7 articles in the RCT subgroup showed low heterogeneity (I²=21%＜50%, and Q test's P=0.27＞0.1), while the 7 articles in the retrospective cohort subgroup exhibited high heterogeneity (I²=90%＞50%, and Q test's P＜0.1). A sensitivity analysis was conducted on the 7 articles in the retrospective cohort subgroup, which indicated that the studies by Shaochun Zhang in 2021 and Guibin Chen in 2022 had a significant impact on the heterogeneity. After excluding these two studies, the remaining 5 articles exhibited low heterogeneity (I²=38%＜50%, P=0.17＞0.1). The overall heterogeneity of the remaining 12 articles after excluding Shaochun Zhang in 2021 and Guibin Chen in 2022 was weak (I²=28%, and Q test's P=0.17＞0.1), and the inter-group heterogeneity was also low (I²=19.5%＜50%, and Q test's P=27＞0.1). Therefore, a fixed-effect model was used for the Meta-analysis.

The pooled mean difference of the overall 12 articles was 1.59, and it was statistically significant (Z=2.88, P＜0.05). Among them, the combined results of 7 RCTs showed a mean difference of 1.3, and it was statistically significant (Z=2.13, P＜0.05), indicating that the AOFAS score at 6 months postoperatively was higher in the elastic fixation group than in the rigid fixation group. Additionally, the combined results of 5 retrospective cohort studies showed a mean difference of 2.90, and it was statistically significant (Z=2.23, P＜0.05), also suggesting that the AOFAS score at 6 months postoperatively was higher in the elastic fixation group than in the rigid fixation group. Details are shown in Figure 6. Overall, based on both the overall analysis and subgroup analyses, the AOFAS score at 6 months postoperatively was slightly higher in the elastic fixation group compared to the rigid fixation group.

Publication bias was examined through the construction of a funnel plot, as depicted in Figure 7. The funnel plot from this study appeared symmetrical, indicating the absence of publication bias in the included literature.


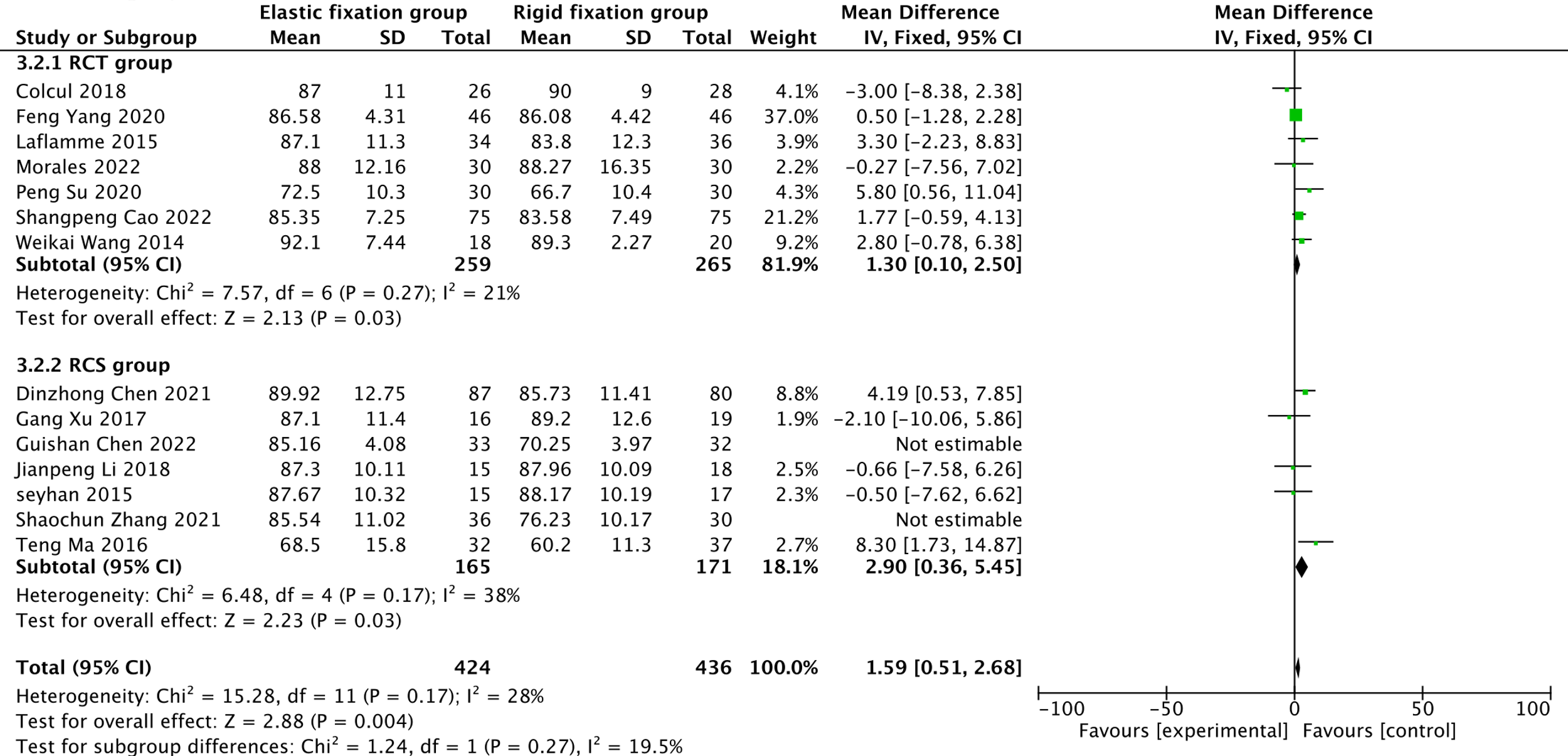


**Figure 6 AOFAS score forest plot 6 months after operation**


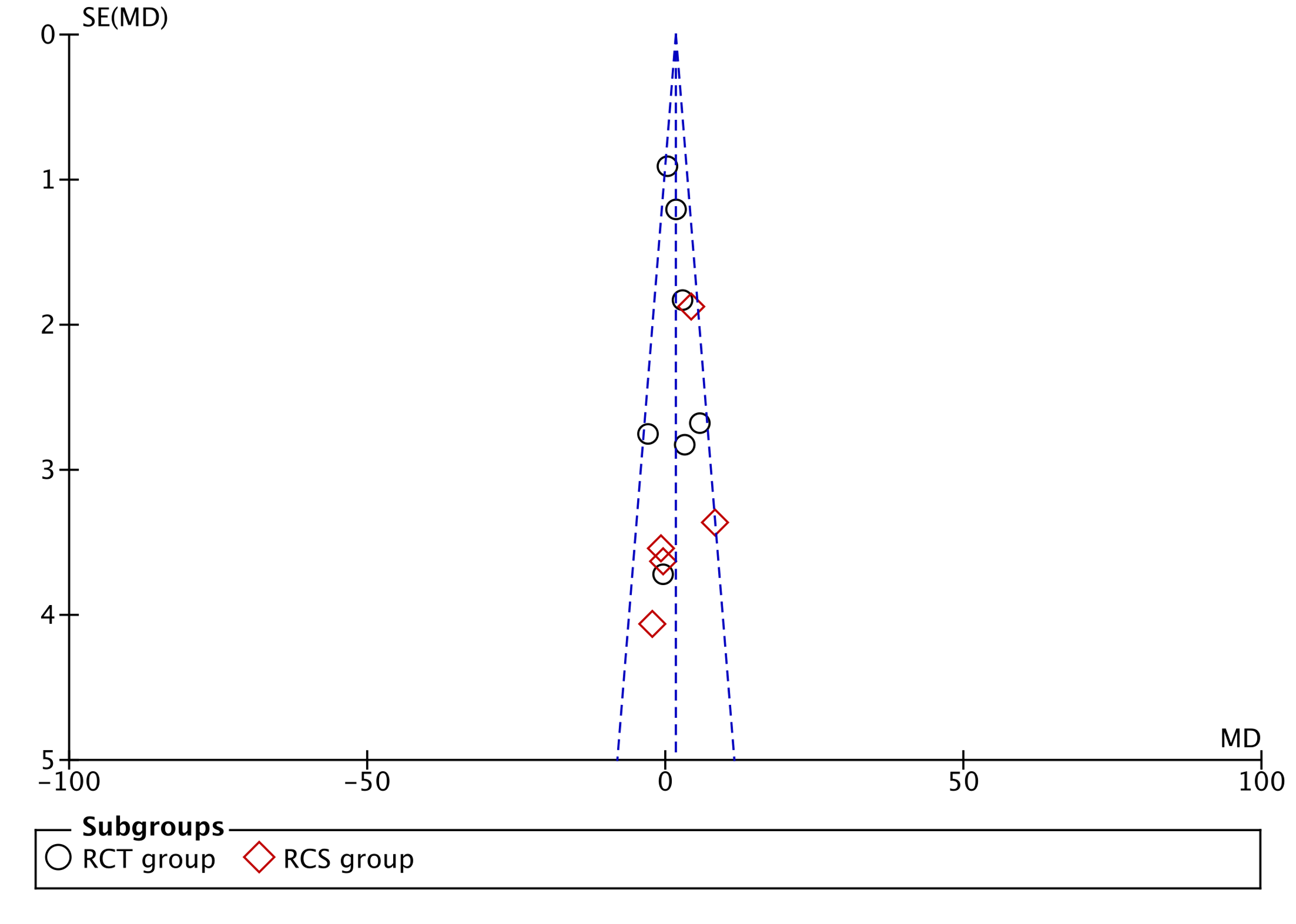


Figure 7 **AOFAS score funnel plot 6 months after operation**

3. AOFAS Scores at 12 Months Postoperatively

A total of 18 articles^[5-8, 10-14, 16, 17, 19-25]^ were included in the analysis of AOFAS scores at 12 months postoperatively. The included literature was divided into subgroups based on the study types: randomized controlled trials (RCTs) and retrospective cohort studies. The heterogeneity test revealed that there was no heterogeneity among the 11 articles in the RCT subgroup (I²=0%, and Q test's P=0.46＞0.1), and the 7 articles in the retrospective cohort subgroup exhibited low heterogeneity (I²=13%＜50%, and Q test's P=0.33＞0.1). Additionally, no significant heterogeneity was observed between the RCT subgroup and the retrospective cohort subgroup (I²=0%, P=0.63＞0.1). Therefore, a fixed-effect model was used for the Meta-analysis.

The pooled mean difference of the overall 18 articles was 2.00, and it was statistically significant (Z=5.46, P＜0.05). Among them, the combined results of 11 RCTs showed a mean difference of 1.92, and it was statistically significant (Z=4.58, P＜0.05), indicating that the AOFAS score at 12 months postoperatively was higher in the elastic fixation group than in the rigid fixation group. Additionally, the combined results of 7 retrospective cohort studies showed a mean difference of 2.25, and it was statistically significant (Z=3.00, P＜0.05), also suggesting that the AOFAS score at 12 months postoperatively was higher in the elastic fixation group than in the rigid fixation group. Details are shown in Figure 8. Overall, based on both the overall analysis and subgroup analyses, the AOFAS score at 12 months postoperatively was higher in the elastic fixation group compared to the rigid fixation group.

Publication bias was examined through the construction of a funnel plot, as depicted in Figure 9. The funnel plot from this study appeared symmetrical, indicating the absence of publication bias in the included literature.


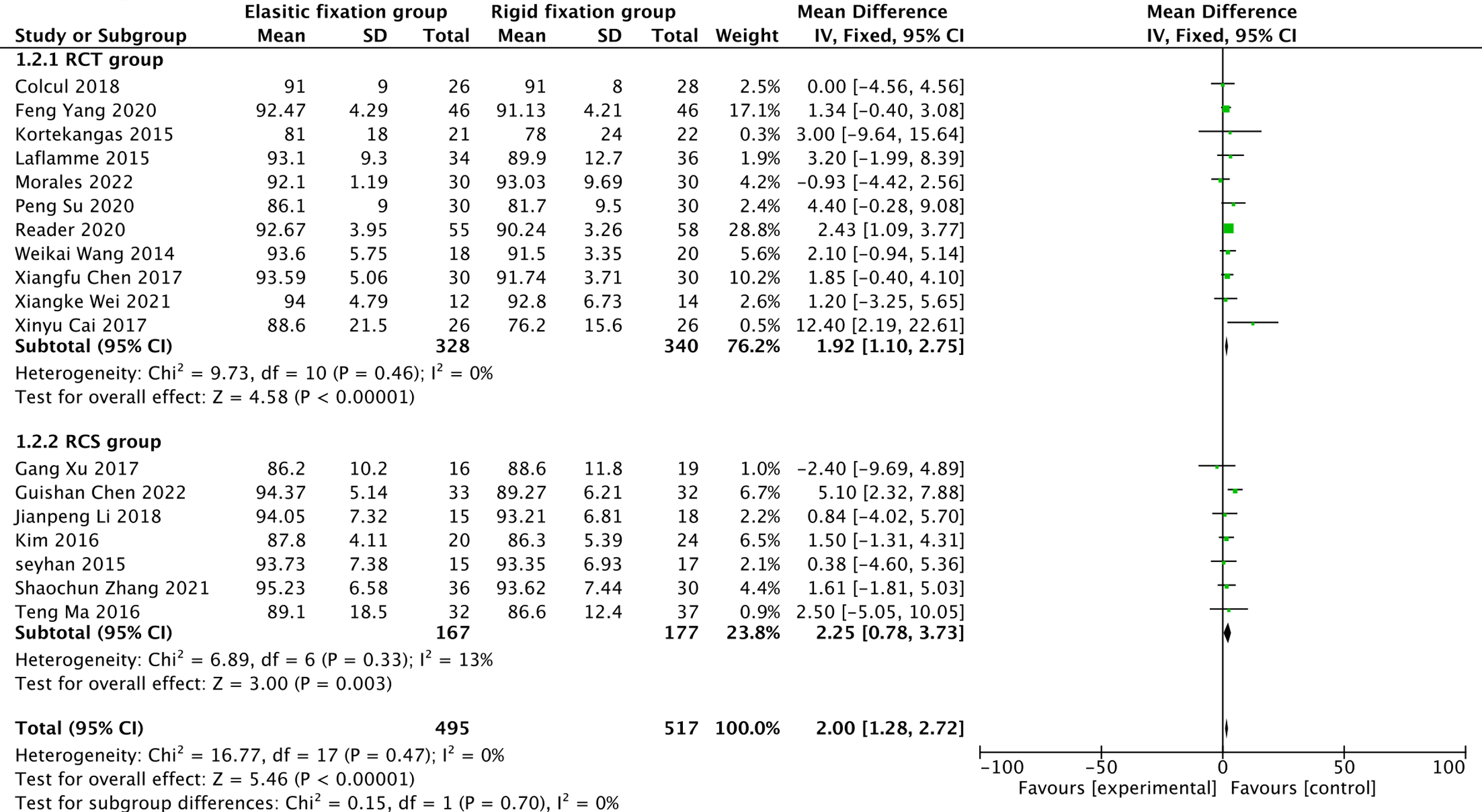


**Figure 8 AOFAS score forest plot 12 months after operation**


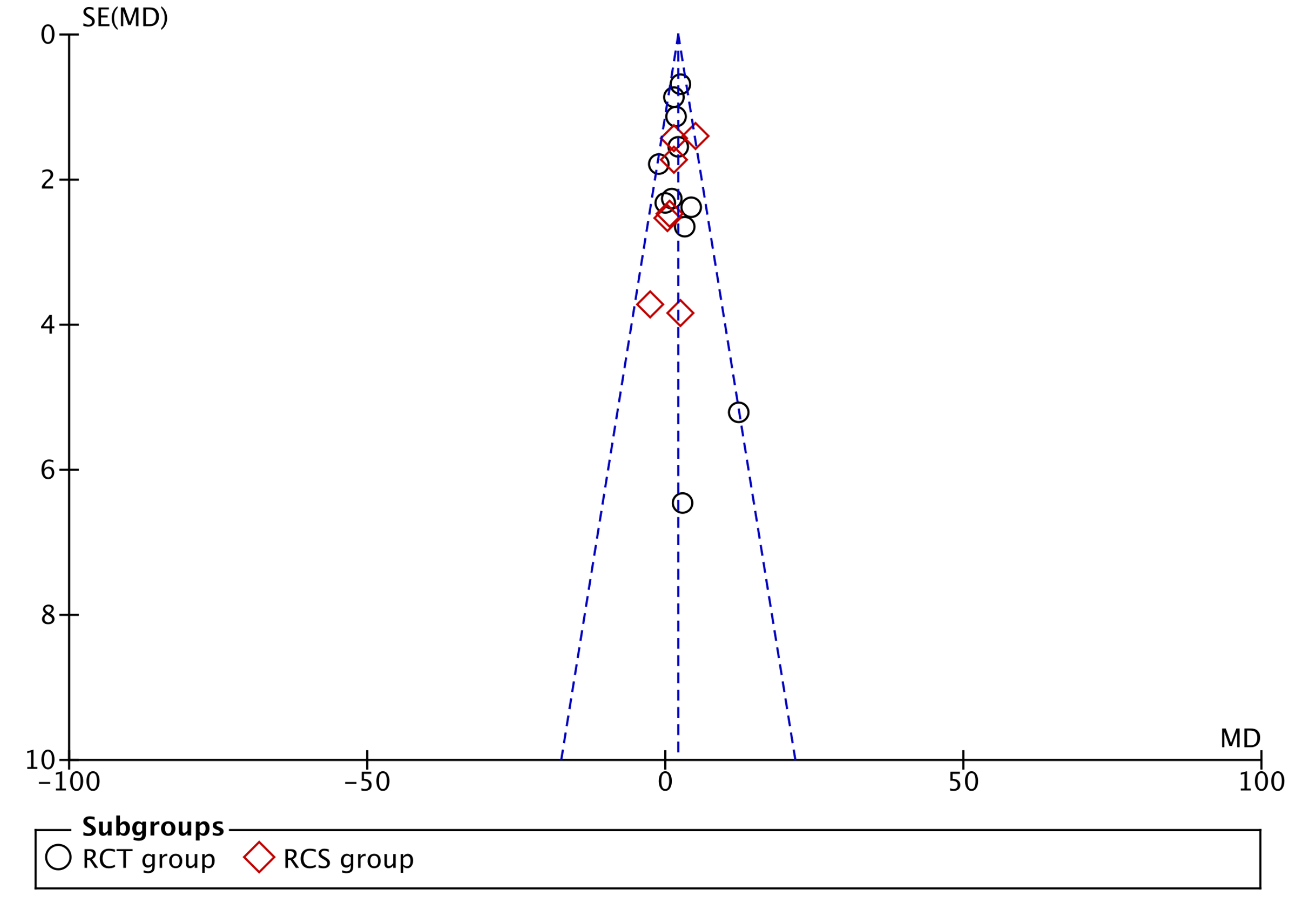


**Figure 9 AOFAS score funnel plot 12 months after operation**

Early Postoperative and Postoperative 12-Month TBCS.

1. Early Postoperative TBCS

Included in the analysis were 11 articles ^[9, 10, 17, 25-32]^ for early postoperative TBCS. Through heterogeneity testing, I²=28%＜50%, and the P value of the Q test is 0.18＞0.1, indicating that the heterogeneity among the selected research articles in this study is not statistically significant. Therefore, a fixed-effect model was used for the Meta-analysis.

The pooled mean difference from the 11 articles is 0.19, with a 95% confidence interval of 0.12 to 0.25, and it is statistically significant. Z=5.64, P＜0.05, suggesting that in the early postoperative period, the tibiofibular syndesmosis gap is smaller in the rigid fixation group compared to the elastic fixation group. For specific details, please refer to Figure 10.

As shown in Figure 11, the funnel plot of this study is symmetric, indicating the absence of publication bias in the included literature.


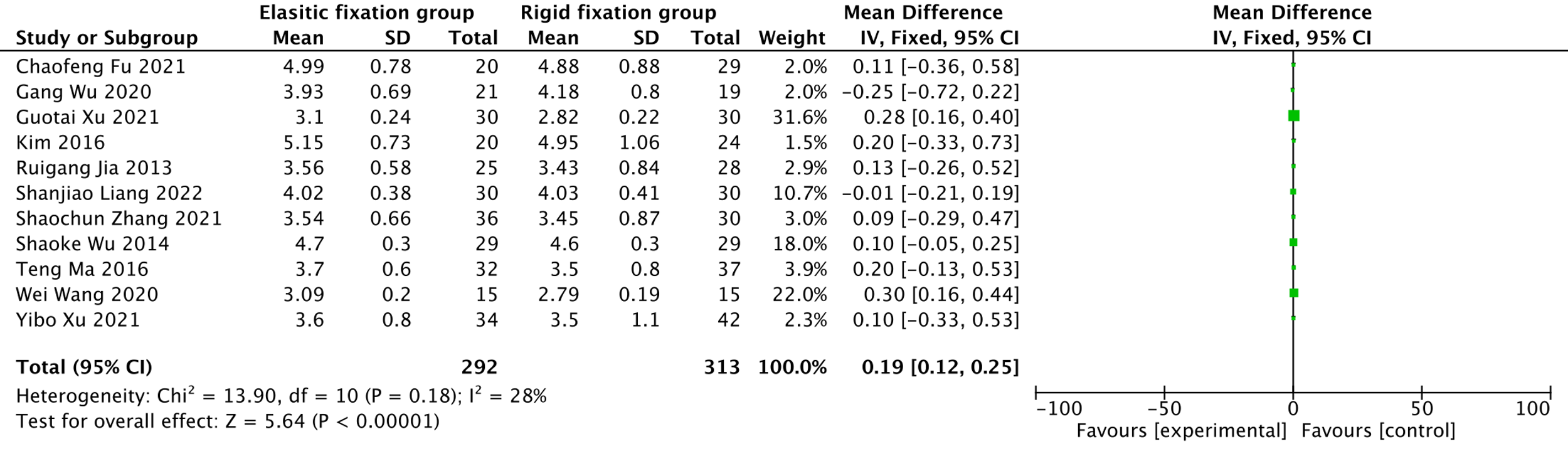


**Figure 10 TBCS forest plot Initial postoperative period**


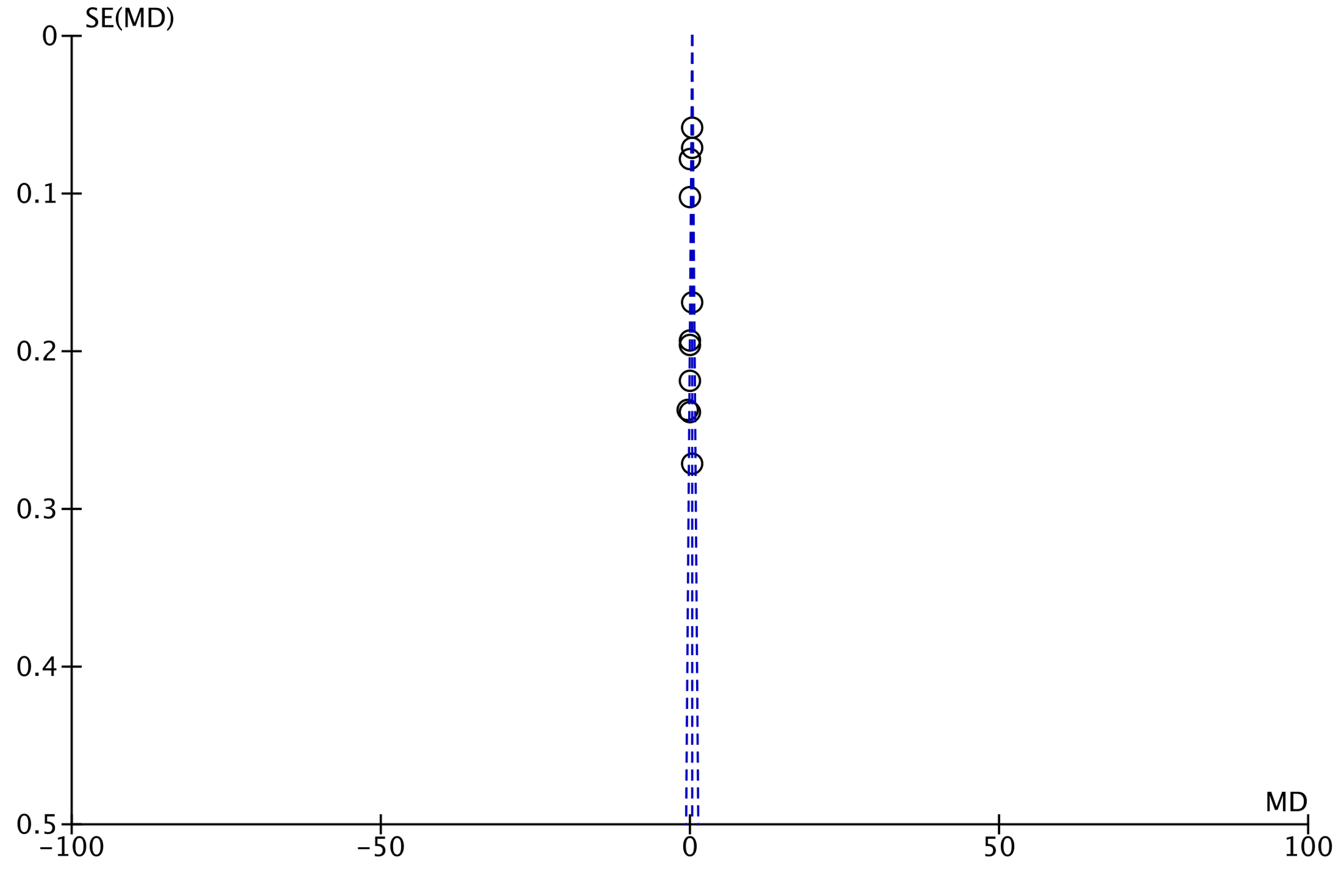


**Figure 11 TBCS funnel plot Initial postoperative period**

2. 12-Month Postoperative TBCS

A total of 11 articles ^[1, 6, 10, 16, 17, 21, 31-35]^ were included for the analysis of 12-month postoperative TBCS. After conducting a heterogeneity test, I²=53%＞50%, and the P value of the Q test is ＜0.1, indicating that the heterogeneity among the selected research articles in this study has statistical significance, warranting a heterogeneity investigation. Sensitivity analysis revealed that the study by Lou Yu Liang in 2016 had a significant impact on the heterogeneity. After excluding this study, the remaining 10 articles showed no statistically significant heterogeneity among them (I²=3%＜50%, and the Q test's P value is 0.41＞0.1). Therefore, after excluding this study, a fixed-effect model was used for further Meta-analysis.

The pooled mean difference from the 10 articles is -0.06, with a P value of 0.12＞0.05. This suggests that the difference in tibiofibular syndesmosis gap between the elastic fixation and rigid fixation groups at 12 months postoperatively is not statistically significant. For specific details, please refer to Figure 12.

As shown in Figure 13, the funnel plot of this study is symmetric, indicating the absence of publication bias in the included literature.


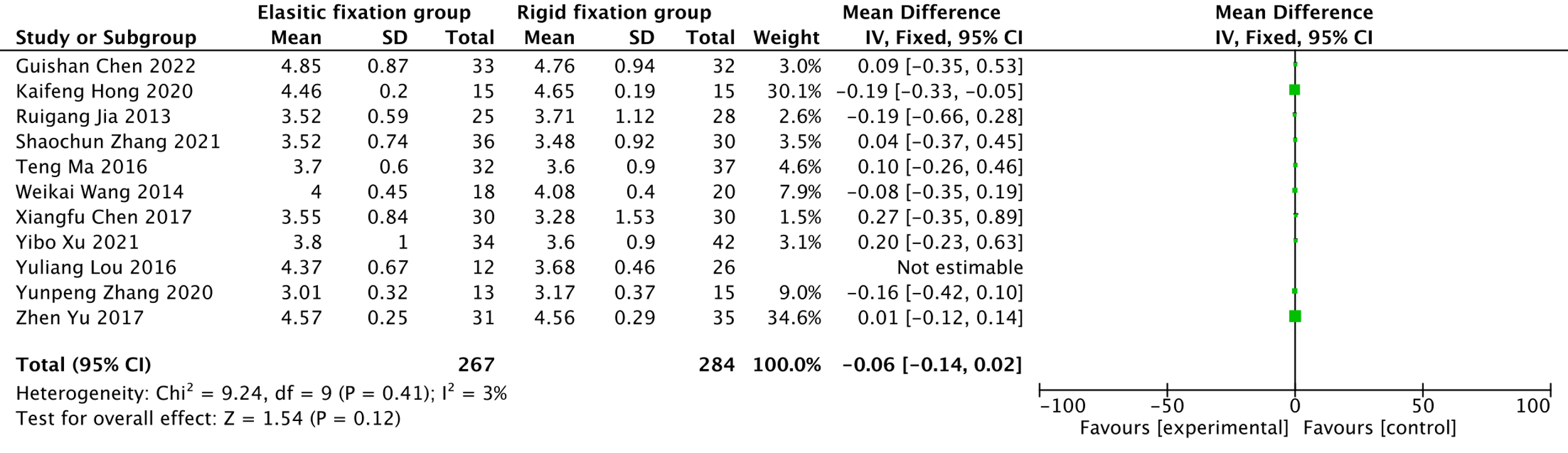


**Figure 12 TBCS forest plot 12 months after operation**

**
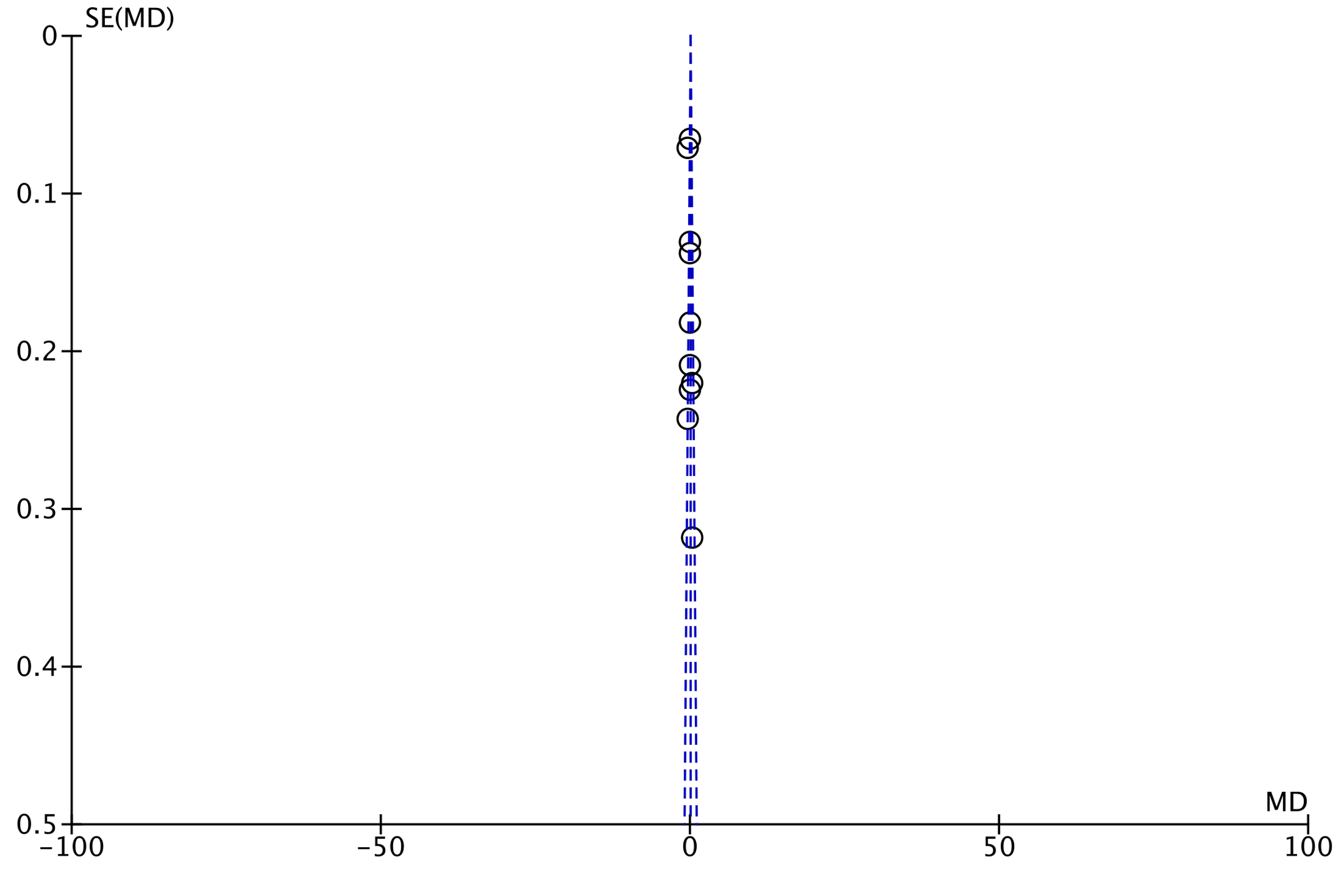
**

**Figure 13 TBCS funnel plot 12 months after operation**

Early Postoperative and Postoperative 12-Month TBOL.

1. Early Postoperative TBOL

A total of 11 articles ^[9, 10, 17, 25-32]^ were included for the analysis of early postoperative TBOL. After conducting a heterogeneity test, I²=38%＜50%, and the P value of the Q test is 0.1, indicating that the heterogeneity among the selected research articles in this study does not have statistical significance. Therefore, a fixed-effect model was used for the analysis.

The pooled mean difference from the 11 articles is 0.06, with a P value of 0.23＞0.05. This suggests that the difference in tibiofibular overlap between the elastic fixation and rigid fixation groups in the early postoperative period is not statistically significant. For specific details, please refer to Figure 14.

As shown in Figure 15, the funnel plot of this study is symmetric, indicating the absence of publication bias in the included literature.


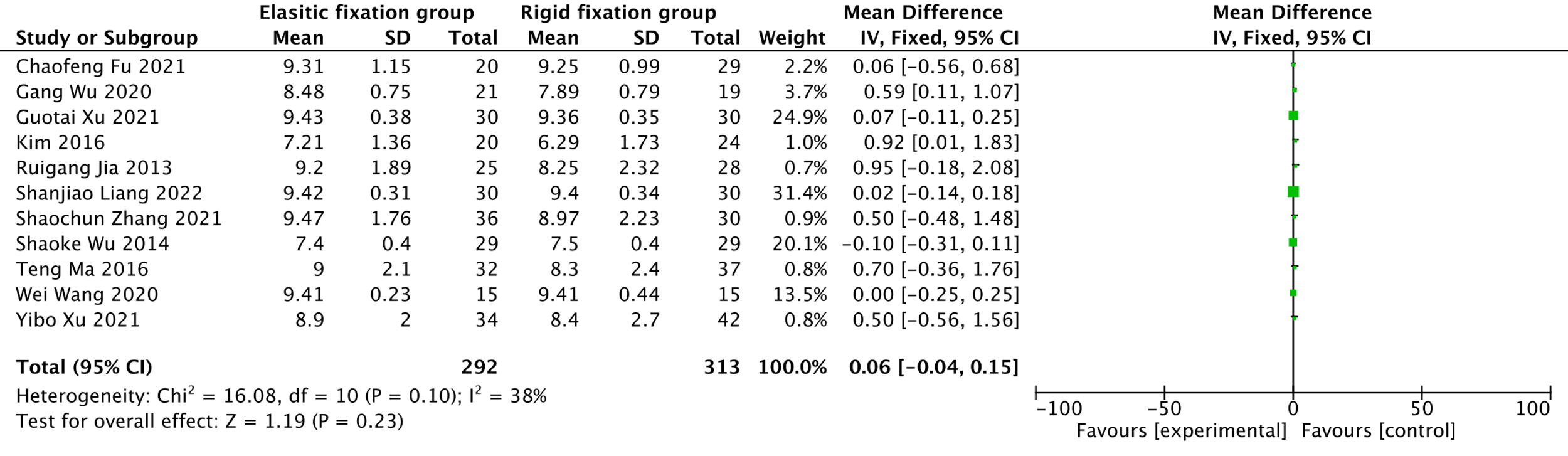


**Figure 14 TBOL forest plot Initial postoperative period**


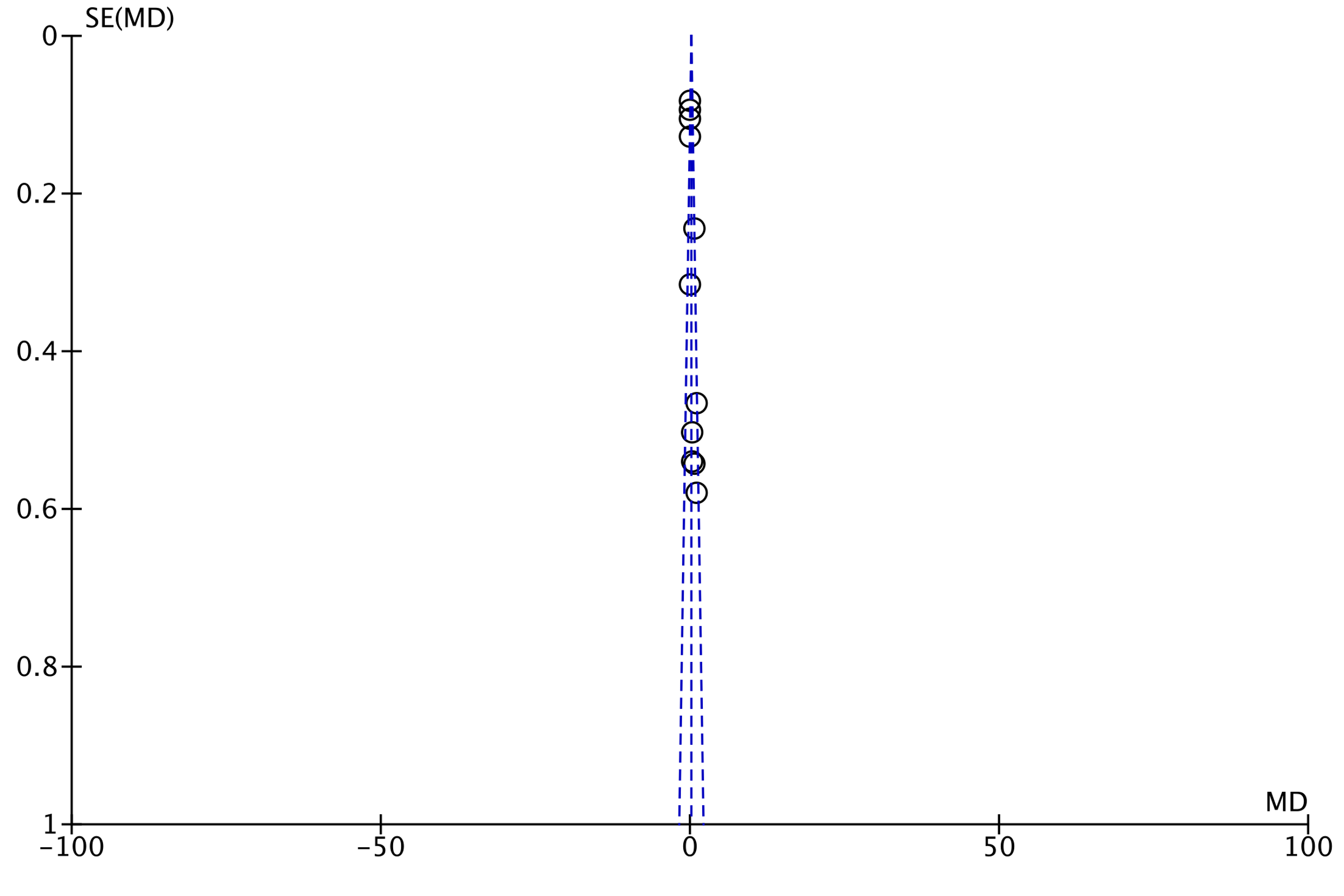


**Figure 15 TBOL funnel plot Initial postoperative period**

2. 12-Month Postoperative TBOL

A total of 11 articles^[1, 6, 10, 16, 17, 21, 31-35]^ were included for the analysis of TBOL at 12 months postoperatively. After conducting a heterogeneity test, I²=25%＜50%, and the P value of the Q test is 0.20＞0.1, indicating that the heterogeneity among the selected research articles in this study does not have statistical significance. Therefore, a fixed-effect model was used for the analysis.

The pooled mean difference from the 11 articles is 0.13, with a 95% confidence interval ranging from 0.03 to 0.24, and it is statistically significant. Z=2.58, with a P value of 0.01＜0.05, suggesting that at 12 months postoperatively, the tibiofibular overlap in the elastic fixation group is greater than that in the rigid fixation group. For specific details, please refer to Figure 16.

As shown in Figure 17, the funnel plot of this study is symmetric, indicating the absence of publication bias in the included literature.


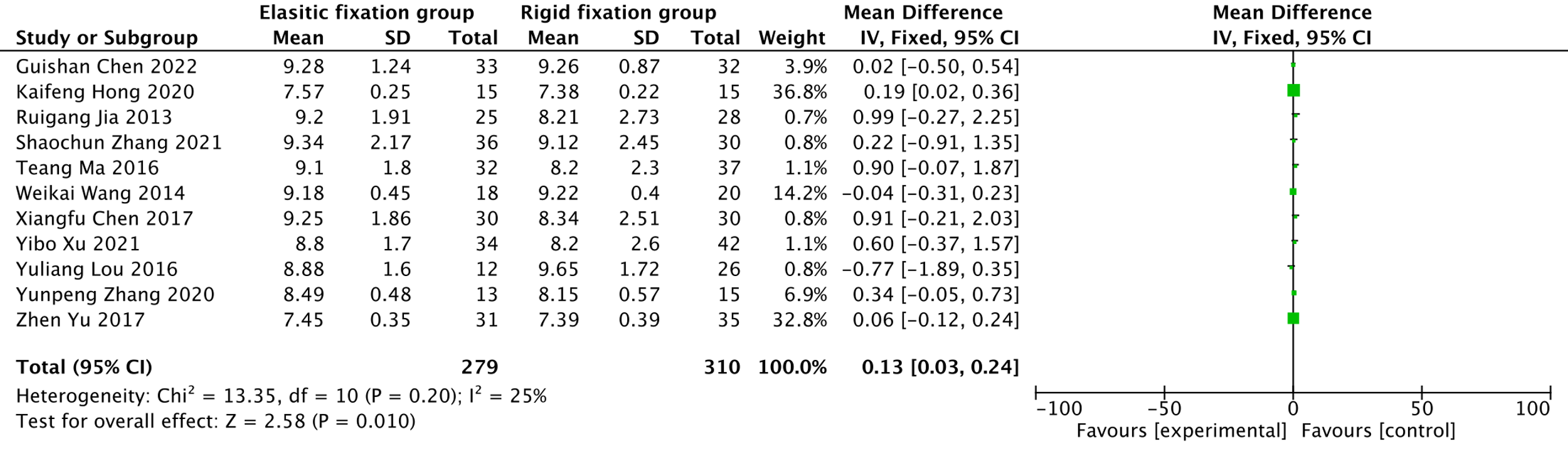


**Figure 16 TBOL forest plot 12 months after operation**


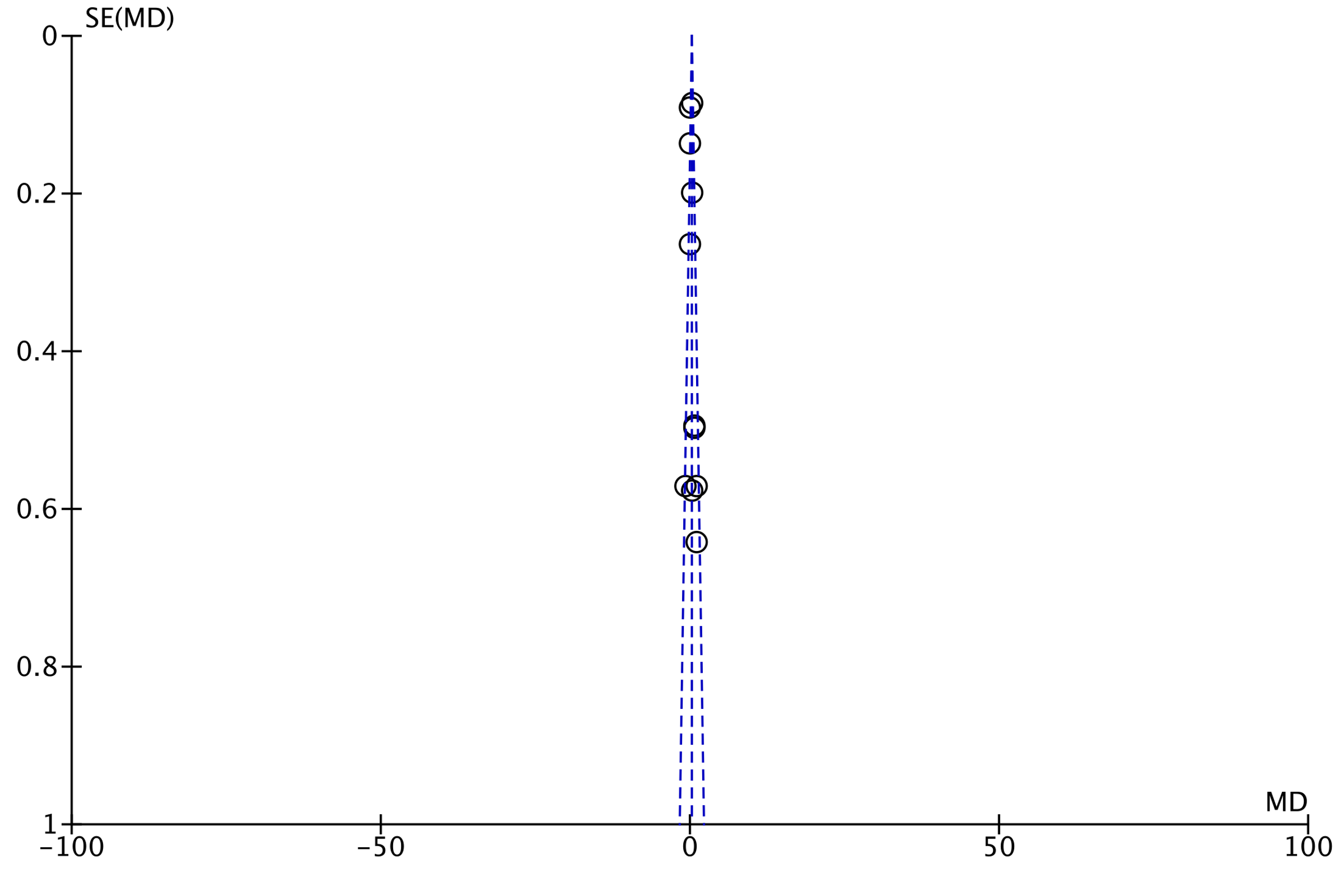


**Figure 17 TBOL funnel plot 12 months after operation**

Postoperative Complications

A total of 13 articles^[9-11, 13-15, 17, 18, 28, 30, 31, 36, 37]^were included for the analysis of postoperative complications, specifically focusing on three aspects: local irritation and wound infection, internal fixation loosening or breakage, and re-displacement of the distal tibia-fibula joint. Through a heterogeneity test, an I² value of 0% and a Q-test's P value of 0.92 (>0.1) were obtained, indicating that the heterogeneity among the selected research articles was not statistically significant. As a result, a fixed-effect model was employed for the analysis .The combined RR value from the 13 articles was 0.41, with a 95% confidence interval ranging from 0.27 to 0.61, and this difference was statistically significant (Z=4.29, P＜0.05). This finding suggests that the incidence of complications in the rigid fixation group was significantly higher than in the elastic fixation group. Please refer to Figure 18 for specific details. The funnel plot in Figure 19 demonstrates symmetry, indicating that there was no publication bias present in this study.

In this aspect, comparing the occurrence rates of local irritation and wound infection between the elastic fixation group and the rigid fixation group, a total of 13 articles^[9-11, 13-15, 17, 18, 28, 30, 31, 36, 37]^were included. Through a heterogeneity test, an I² value of 9% (<50%), and a Q-test's P value of 0.35 (>0.1) were obtained, indicating that the heterogeneity among the selected research articles was not statistically significant. Thus, a fixed-effect model was employed for the analysis. The pooled RR value from the 13 articles was 0.54, with a 95% confidence interval ranging from 0.32 to 0.93, and this difference was statistically significant (Z=2.24, P=0.03 < 0.05). This suggests that the postoperative incidence rate of local irritation and wound infection in the rigid fixation group was significantly higher than in the elastic fixation group. Detailed results can be observed in Figure 20. The symmetric funnel plot in Figure 21 indicates the absence of publication bias in this aspect of the study.

Within the scope of this investigation, a meticulous comparative assessment was conducted to discern variations in the occurrence rates of fixation loosening or fracture between the cohorts subjected to elastic fixation and rigid fixation methodologies. The analyzed dataset comprised thirteen pertinent research articles ^[9-11, 13-15, 17, 18, 28, 30, 31, 36, 37]^, and a robust scrutiny of heterogeneity was undertaken. The resultant I² index of 0% conveys that the dispersion observed among the selected studies does not surpass the conventional threshold of statistical significance (<50%). Furthermore, the Q-test yielded a p-value of 0.98—surpassing the predetermined alpha threshold of 0.1, affirming the lack of noteworthy heterogeneity among the chosen studies. Consequently, the adoption of a fixed-effects model was considered appropriate to facilitate an unbiased and comprehensive analysis. Pooling the outcomes derived from the synthesis of these studies yielded a relative risk (RR) of 0.26, supported by a 95% confidence interval spanning 0.12 to 0.55. The statistical significance of this outcome was corroborated by a Z-score of 3.53, resulting in a p-value of less than 0.05. These findings unequivocally point to a robust statistical indication that the rigid fixation group exhibits a significantly elevated incidence rate of fixation loosening or fracture when compared to the elastic fixation group. These intricate findings have been graphically elucidated in Figure 22. Additionally, the corresponding funnel plot, depicted in Figure 23, is symmetric, further affirming the absence of publication bias and underscoring the reliability of the present study's findings.

This investigation delved into the realm of postoperative tibiofibular diastasis, undertaking a meticulous examination of the divergence in incidence rates between the cohorts subjected to elastic fixation and rigid fixation methodologies. The cohort for analysis encompassed a corpus of research articles totaling thirteen in number [9-11, 13-15, 17, 18, 28, 30, 31, 36, 37]. A comprehensive assessment of heterogeneity was executed, as denoted by the I² statistic. The calculated I² value of 0% signified that the variability observed among the enlisted studies did not exceed the conventional threshold for statistical significance (<50%). Furthermore, the p-value yielded by the Q-test, amounting to 0.92, exceeded the pre-established significance threshold of 0.1. This convergence of results underscored the absence of statistically meaningful heterogeneity among the selected studies, justifying the utilization of the fixed-effects model for subsequent analysis. The amalgamation of results across the studies yielded a relative risk (RR) value of 0.74, accompanied by a computed p-value of 0.53—surpassing the alpha threshold of 0.05. This statistical analysis indicates that the variation in the rates of postoperative tibiofibular diastasis between the elastic fixation and rigid fixation groups does not achieve a level of statistical significance. A visual representation of these findings is offered in Figure 24. Furthermore, the funnel plot depicted in Figure 25 demonstrates a symmetrical distribution, affirming the absence of publication bias and bolstering the credibility of the study's findings.

In summary, postoperative complications were observed to be significantly higher in the rigid fixation group as compared to the elastic fixation group. This discrepancy primarily manifested in the occurrence rates of local irritation and wound infection, as well as internal fixation loosening or fracture, which were notably elevated in the rigid fixation cohort when contrasted with the elastic fixation cohort. However, no substantial disparity in the incidence rate of tibiofibular diastasis recurrence was evident between the two groups.


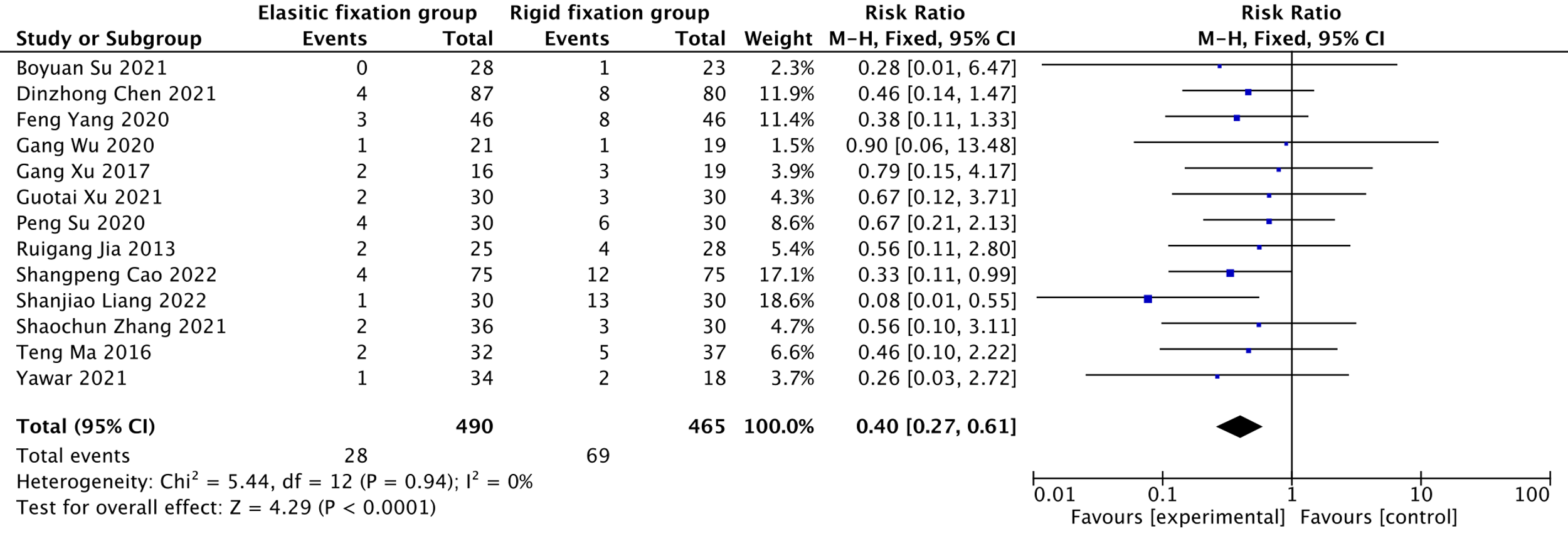


**Figure 18 Forest plot of overall postoperative complications**


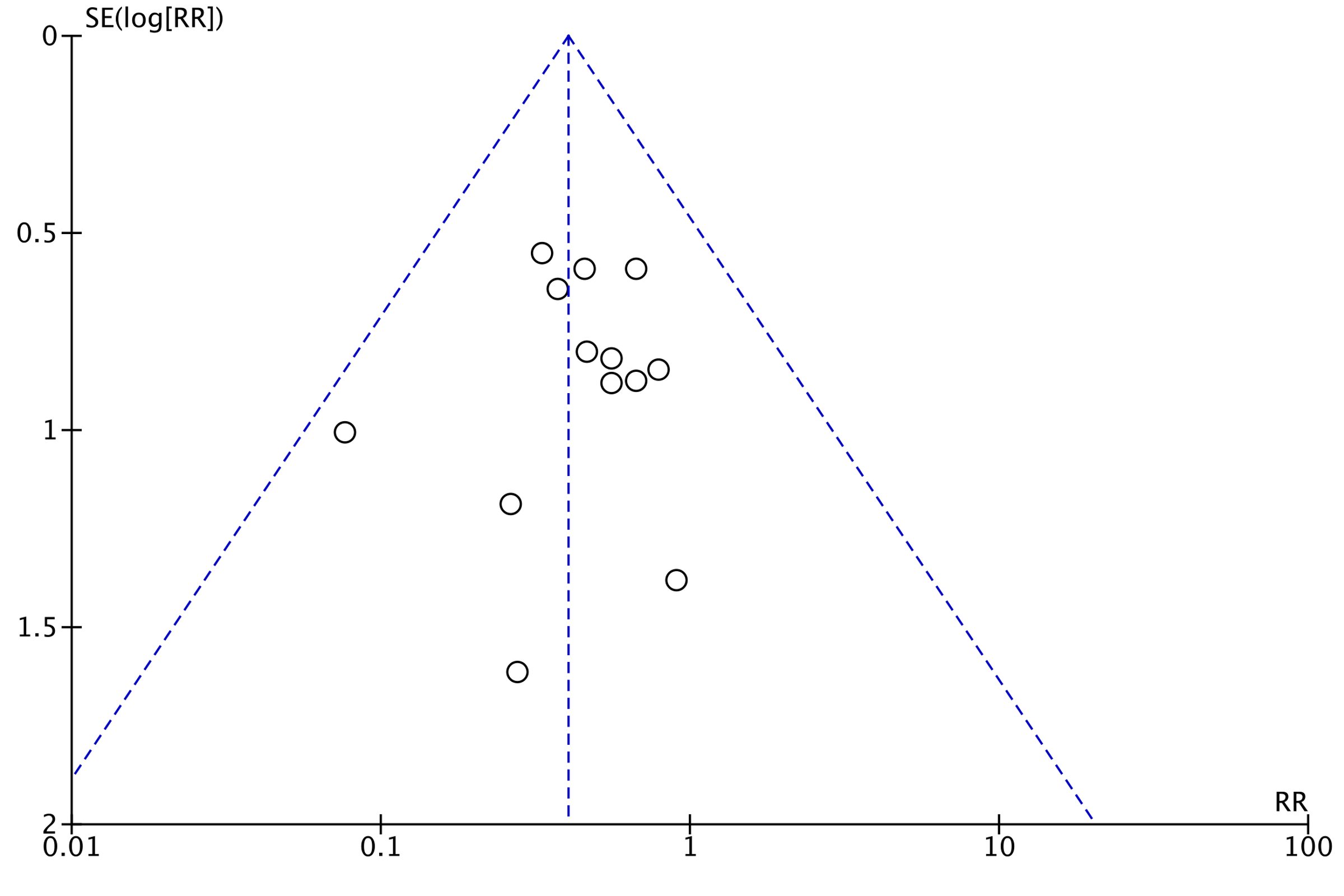


**Figure 19 Funnel plot of overall postoperative complications**


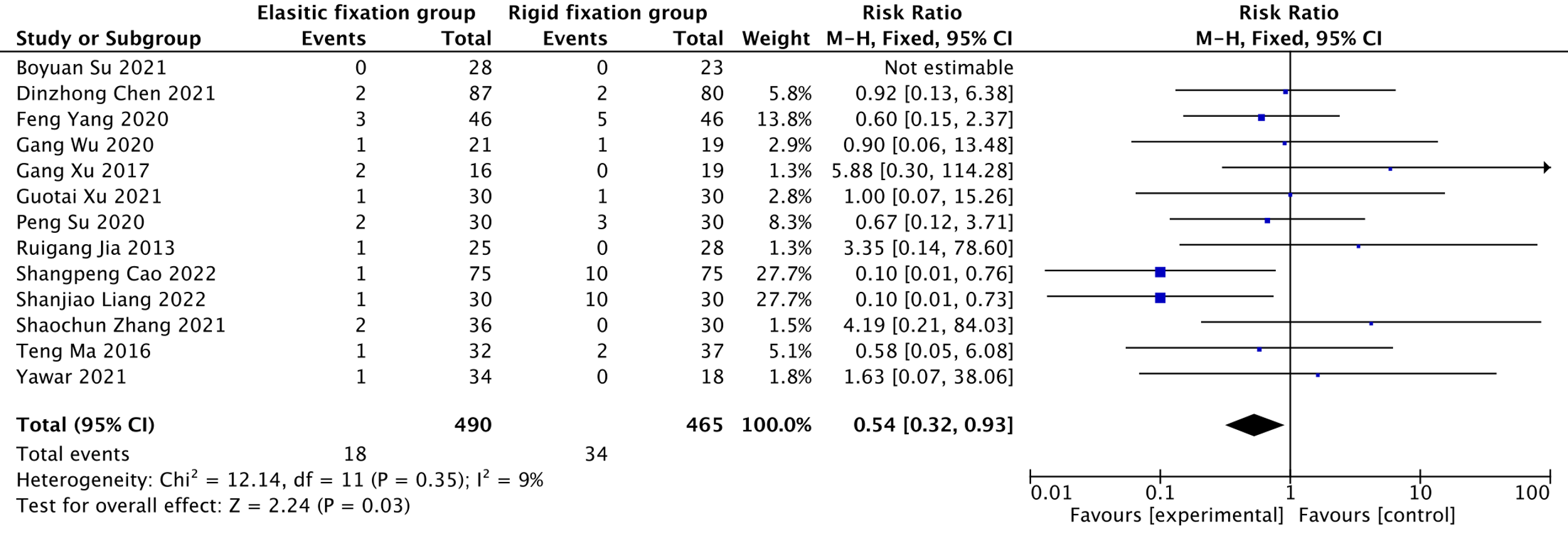


**Figure 20 Forest plot of postoperative local irritation and wound infection**


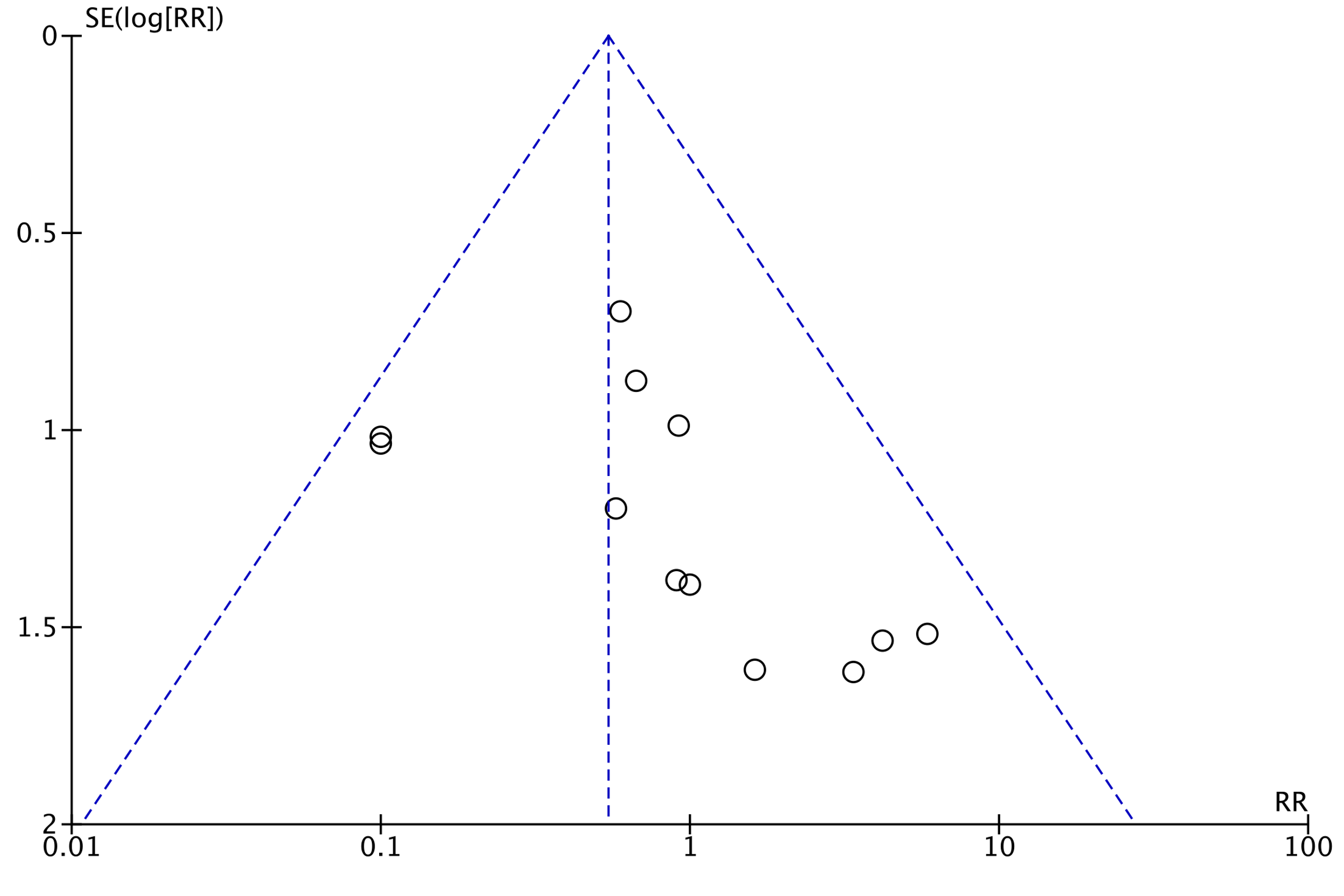


**Figure 21 Funnel plot of postoperative local irritation and wound infection**


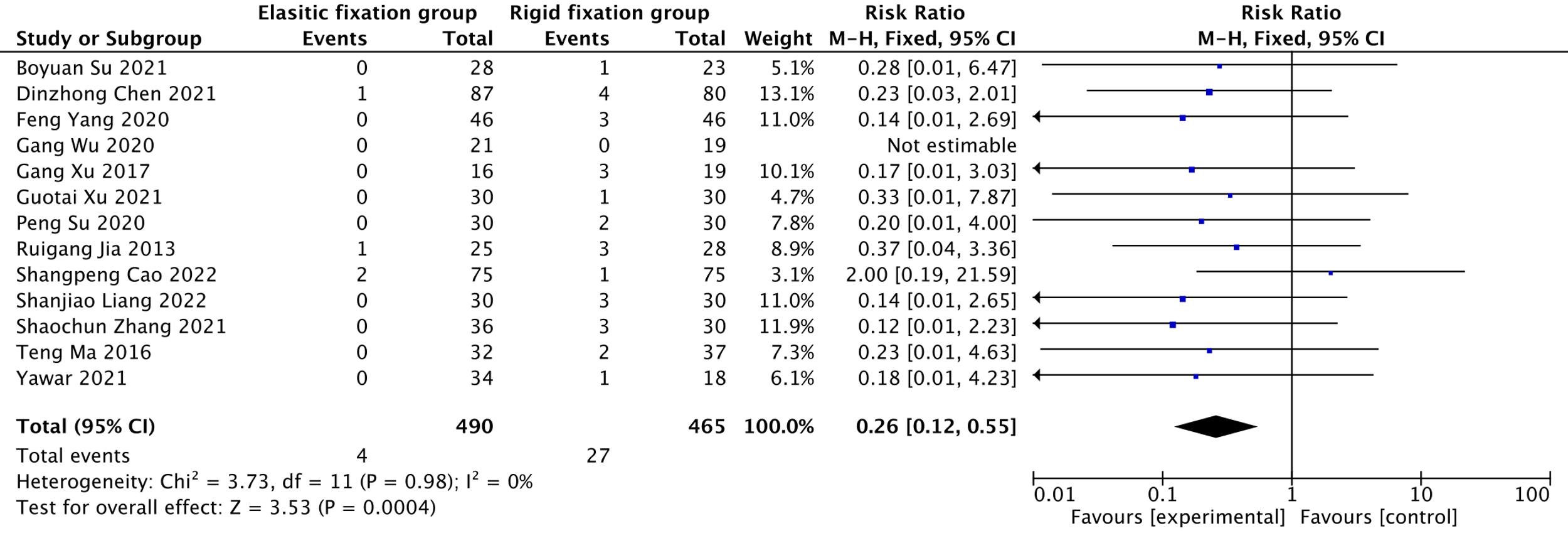


**Figure 22 Forest plot of loosening and fracture of postoperative internal fixation**


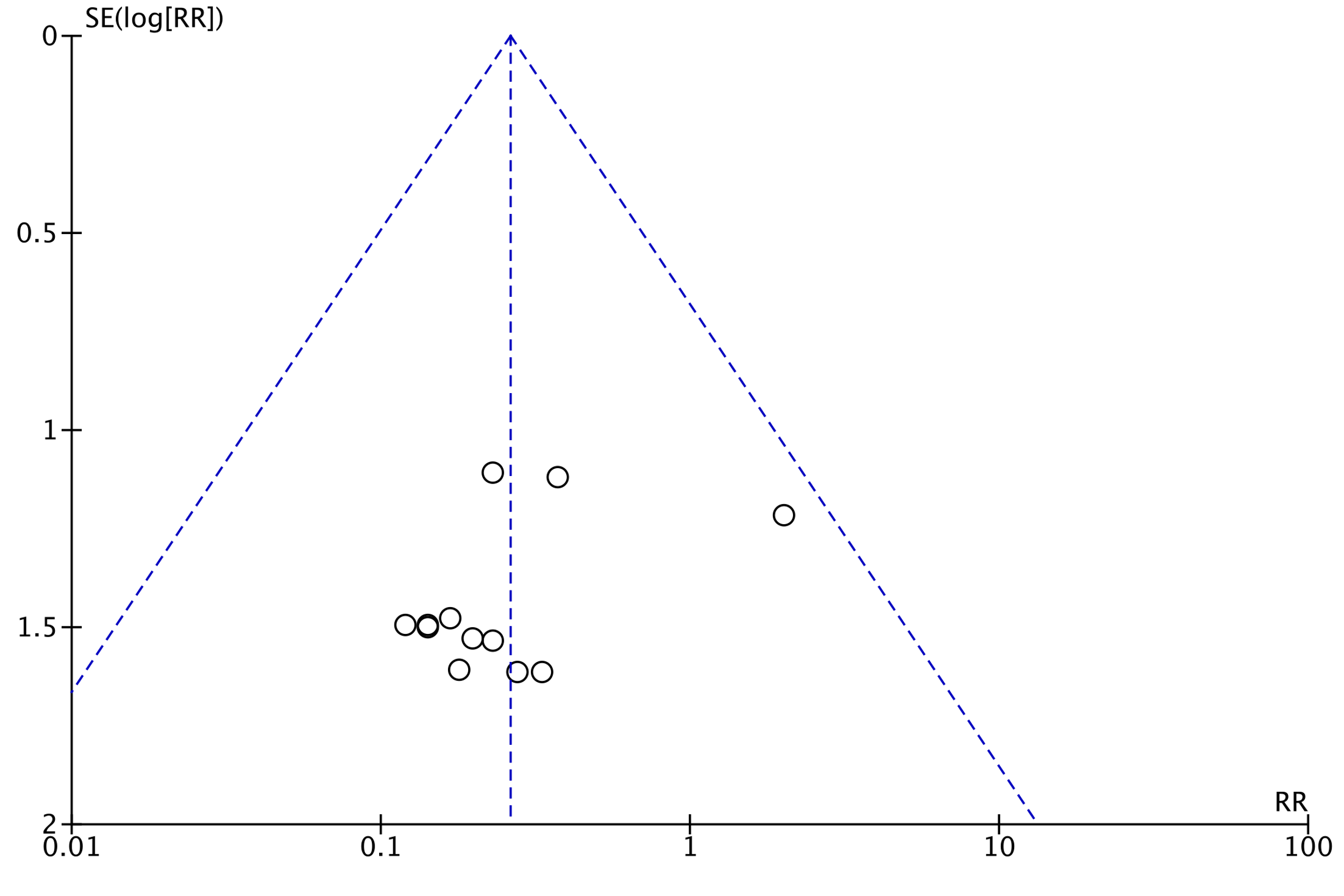


**Figure 23 Funnel plot of loosening and fracture of postoperative internal fixation**


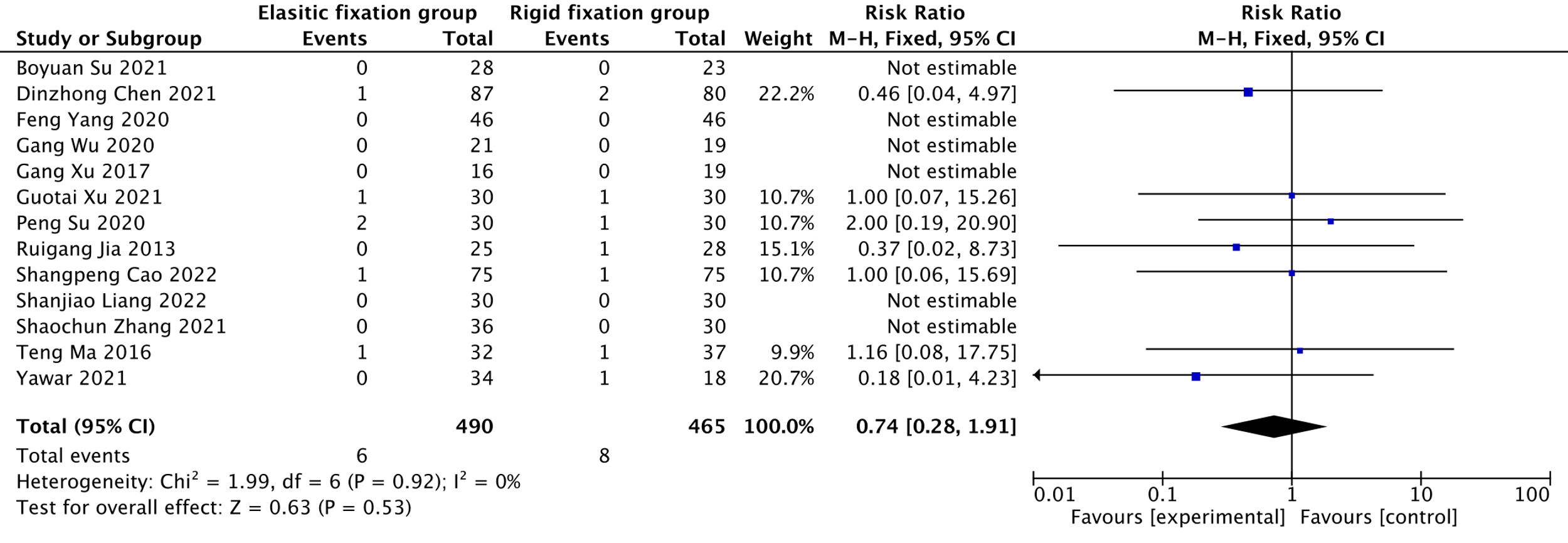


**Figure 24 Forest plot of lower tibiofibular resegregation after surgery**


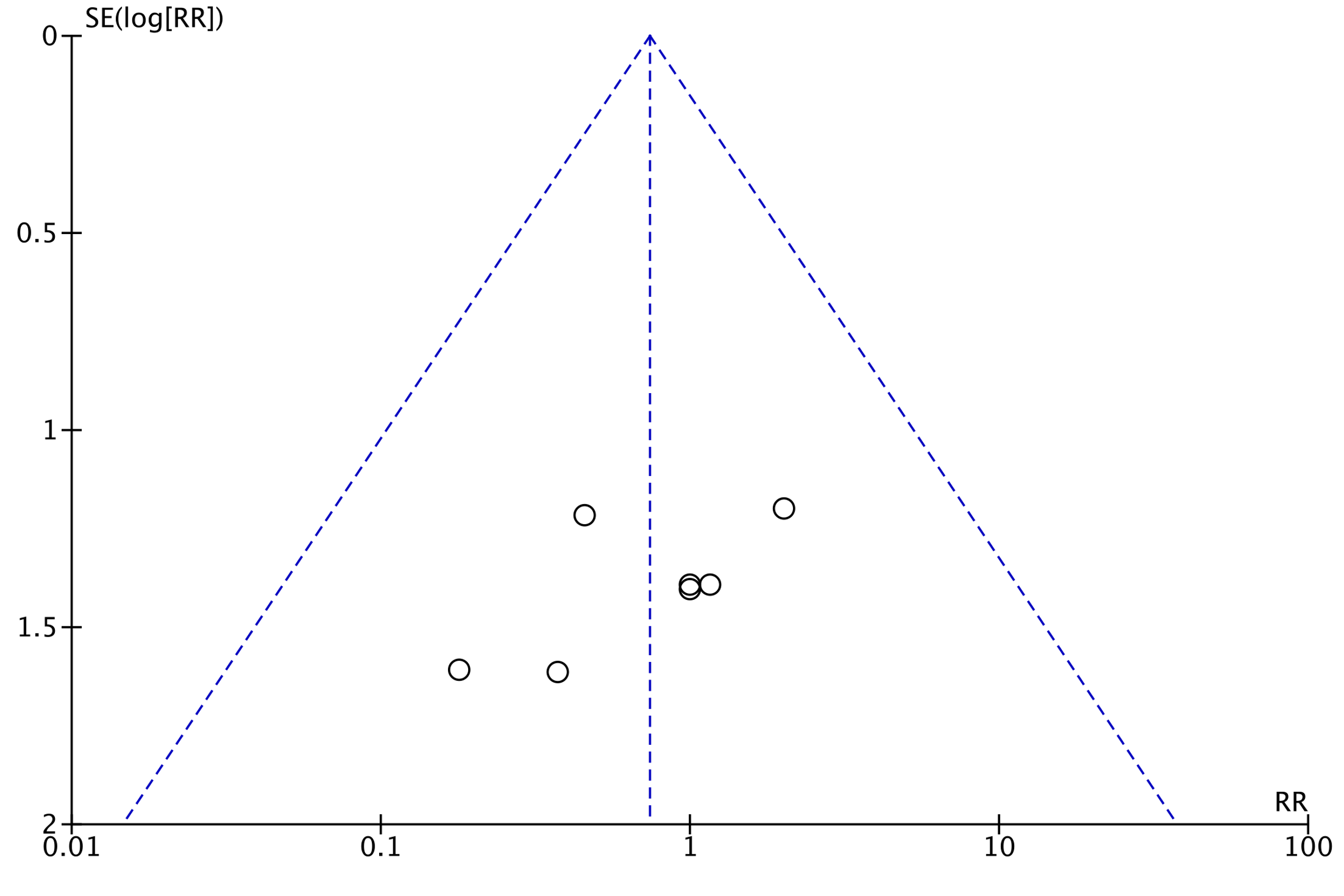


**Figure 25 Funnel plot of lower tibiofibular resegregation after surgery**

Postoperative Full Weight-Bearing Time

Incorporating 11 studies[9, 10, 13, 15, 17, 18, 28-30, 34, 36] , the analysis was conducted to assess the time required for full weight-bearing postoperatively. Heterogeneity testing revealed significant heterogeneity with an I² of 94%, exceeding the threshold of 50%, and a Q-test p-value below 0.1, indicating statistically significant heterogeneity among the selected studies. Given that individual factors greatly influence the time to achieve full weight-bearing after surgery and this heterogeneity cannot be eliminated, a random-effects model was employed for analysis. The pooled analysis of the 11 studies yielded a mean difference of -1.65 with a 95% confidence interval ranging from -2.39 to -0.92, signifying statistical significance. The Z-score was 4.41 (p < 0.05), as depicted in Figure 26. These findings suggest that the time required for complete weight-bearing in the rigid fixation group is greater than that in the elastic fixation group. The funnel plot, illustrated in Figure 27, visually confirms symmetry and indicates the absence of publication bias in this study.


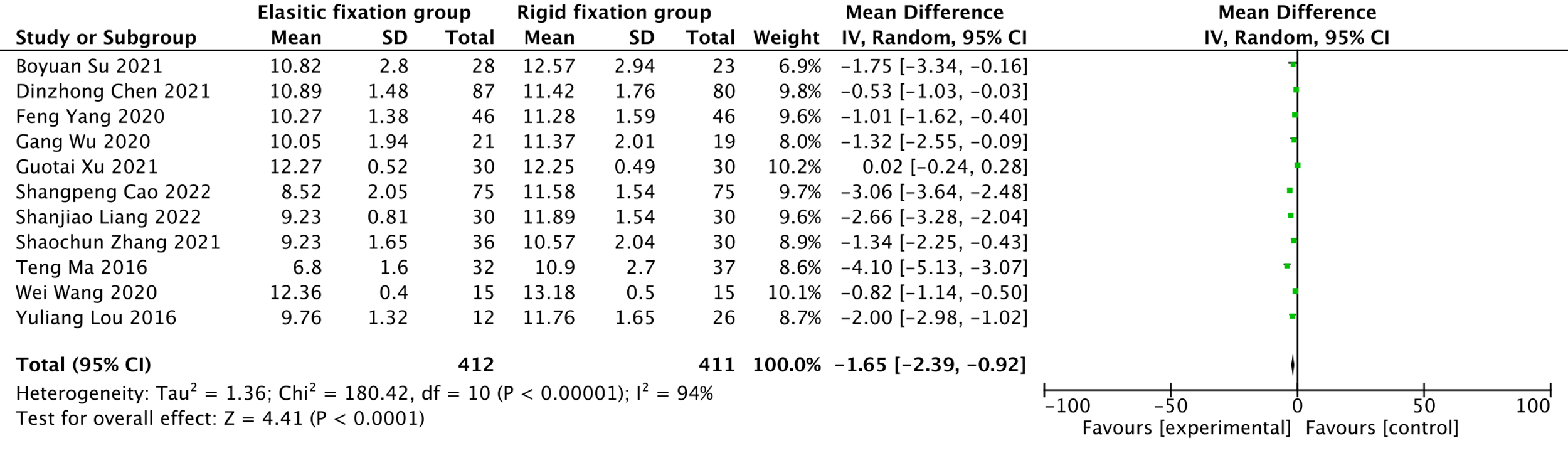


**Figure 26 Forest plot of total weight bearing time after operation**


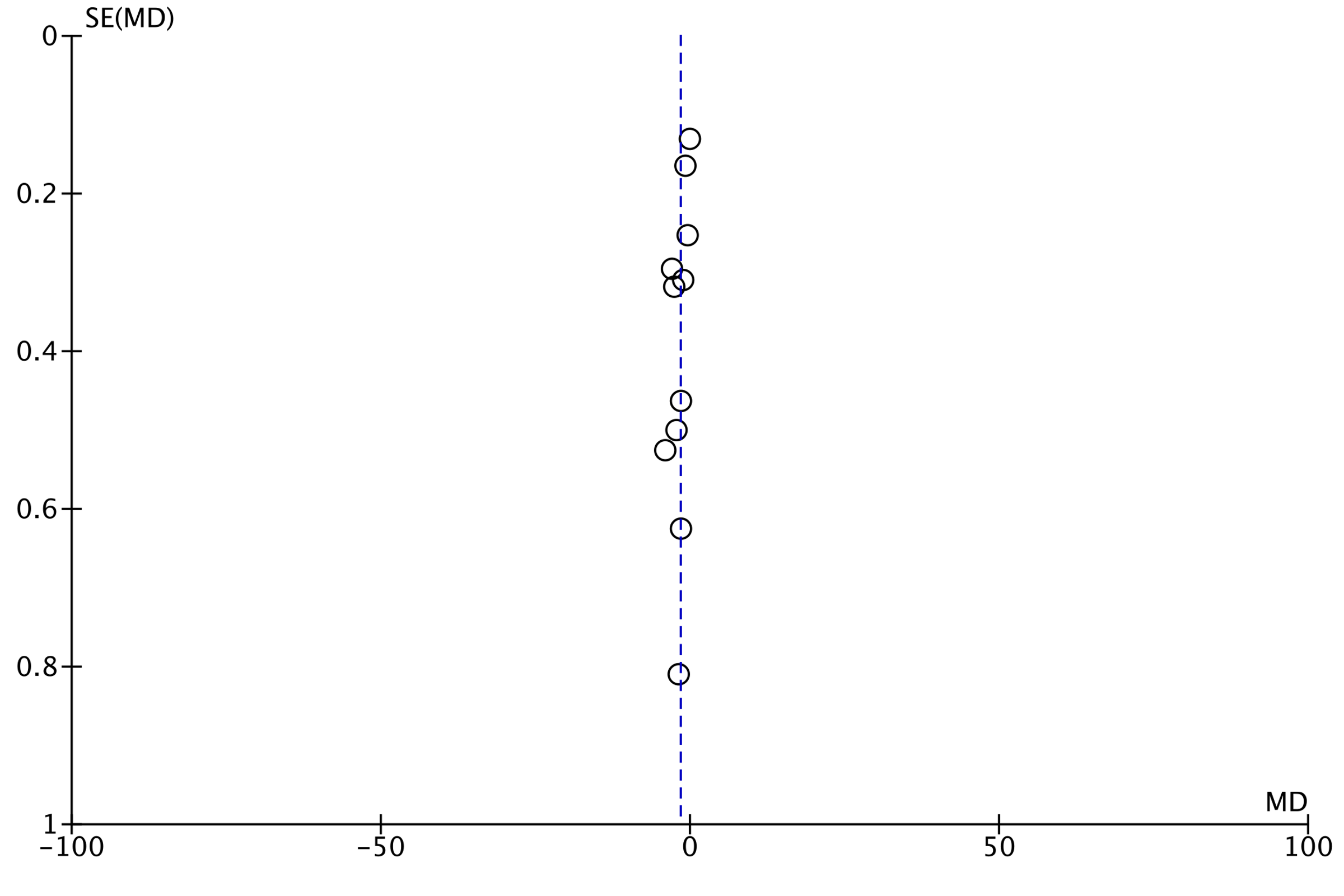


**Figure 27 Funnel plot of total weight bearing time after operation**

Intraoperative Blood Loss and Surgical Duration

1. Intraoperative Blood Loss

Incorporating 13 articles ^[9, 12, 15, 18, 24, 26-28, 30, 33-35, 38]^ for intraoperative blood loss analysis, a funnel plot was constructed, revealing publication bias in the article by Chaofeng Fu 2021. Therefore, excluding this article, the remaining 12 articles were subjected to analysis. Heterogeneity test yielded I²=0%, and the Q-test's P=0.72＞0.1, indicating that the selected research articles did not exhibit statistically significant heterogeneity, warranting the use of a fixed-effects model. The pooled mean difference for the 12 articles was -0.73, with a 95% confidence interval of -1.76 to 0.31. However, as P=0.17＞0.05, no statistical significance was observed, as depicted in Figure 28. This suggests that there is no statistically significant difference in intraoperative blood loss between the elastic fixation group and the rigid fixation group. The funnel plot, displayed in Figure 29, indicates a symmetrical distribution, implying the absence of publication bias in this study.

2. Surgical Duration

Incorporating 12 studies ^[12, 15, 18, 24, 26-28, 30, 33-35, 38]^, surgical duration was subjected to analysis. The heterogeneity test yielded a substantial I² value of 96%, and the p-value from the Q-test was less than 0.1, indicating significant heterogeneity among the selected studies. Given the considerable diversity in surgical experience and practices among individual surgeons, the heterogeneity among the included studies could not be eliminated. Consequently, a random-effects model was employed for the synthesis.

The pooled mean difference of the 12 studies was 9.24, with a 95% confidence interval ranging from 2.91 to 15.57. The results were statistically significant, with a Z-score of 2.86 and a p-value of 0.004 (<0.05), as depicted in Figure 30. This suggests that the surgical duration in the elastic fixation group was slightly longer than that in the rigid fixation group. The funnel plot, presented in Figure 31, demonstrates a symmetrical distribution, indicating the absence of publication bias in this study.


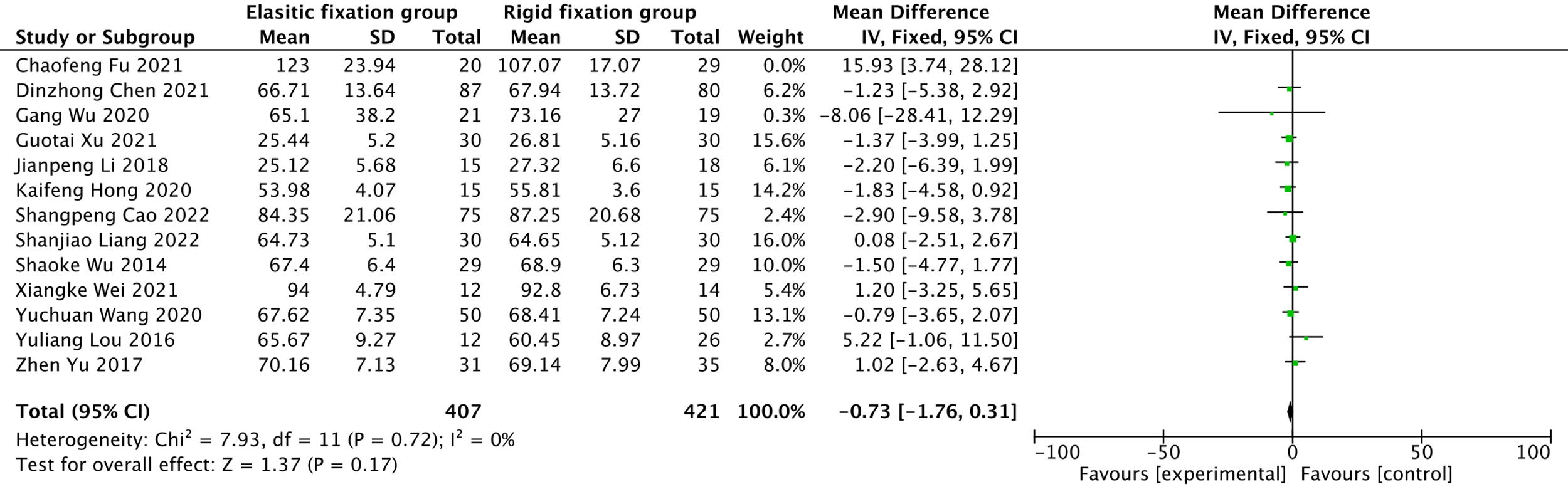


**Figure 28 Forest plot of intraoperative blood loss**


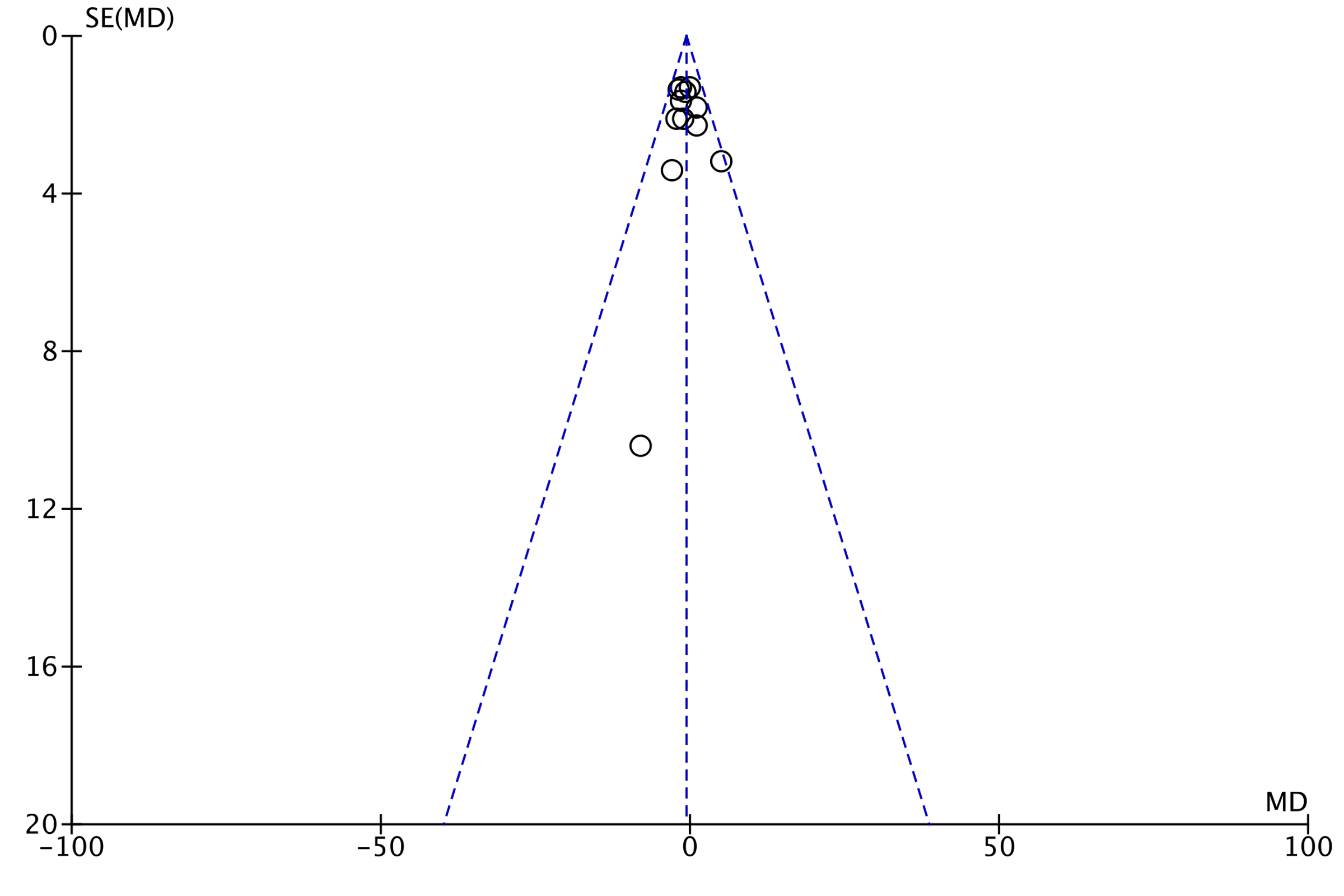


**Figure 29 Funnel plot of intraoperative blood loss**


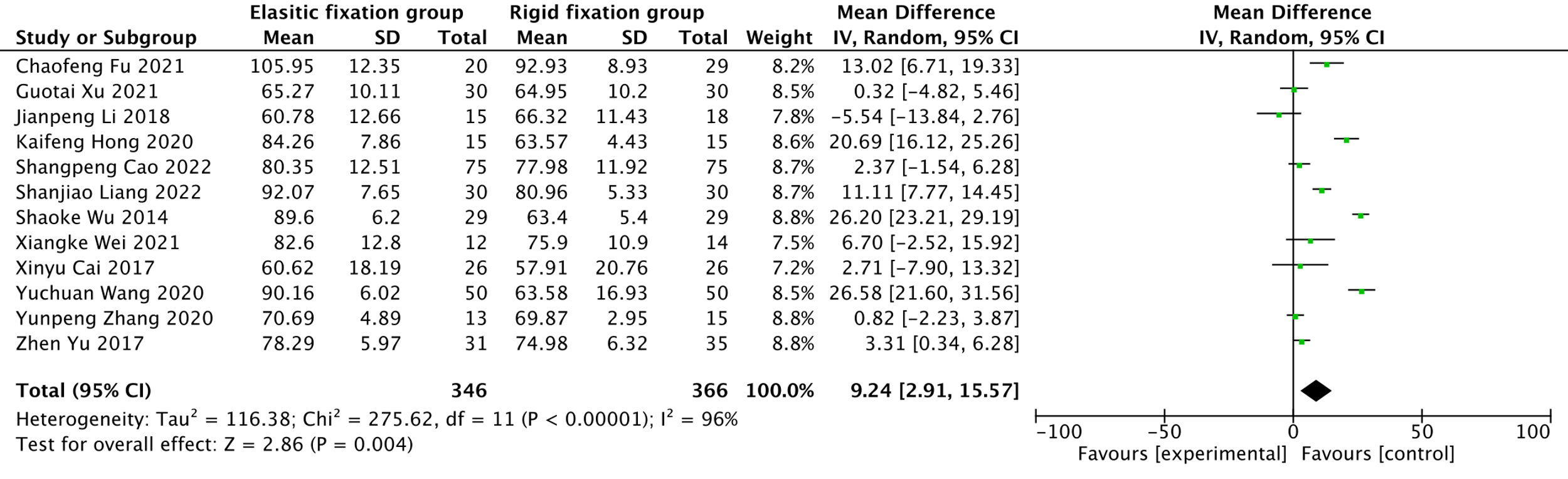


**Figure 30 Forest plot of operative time**


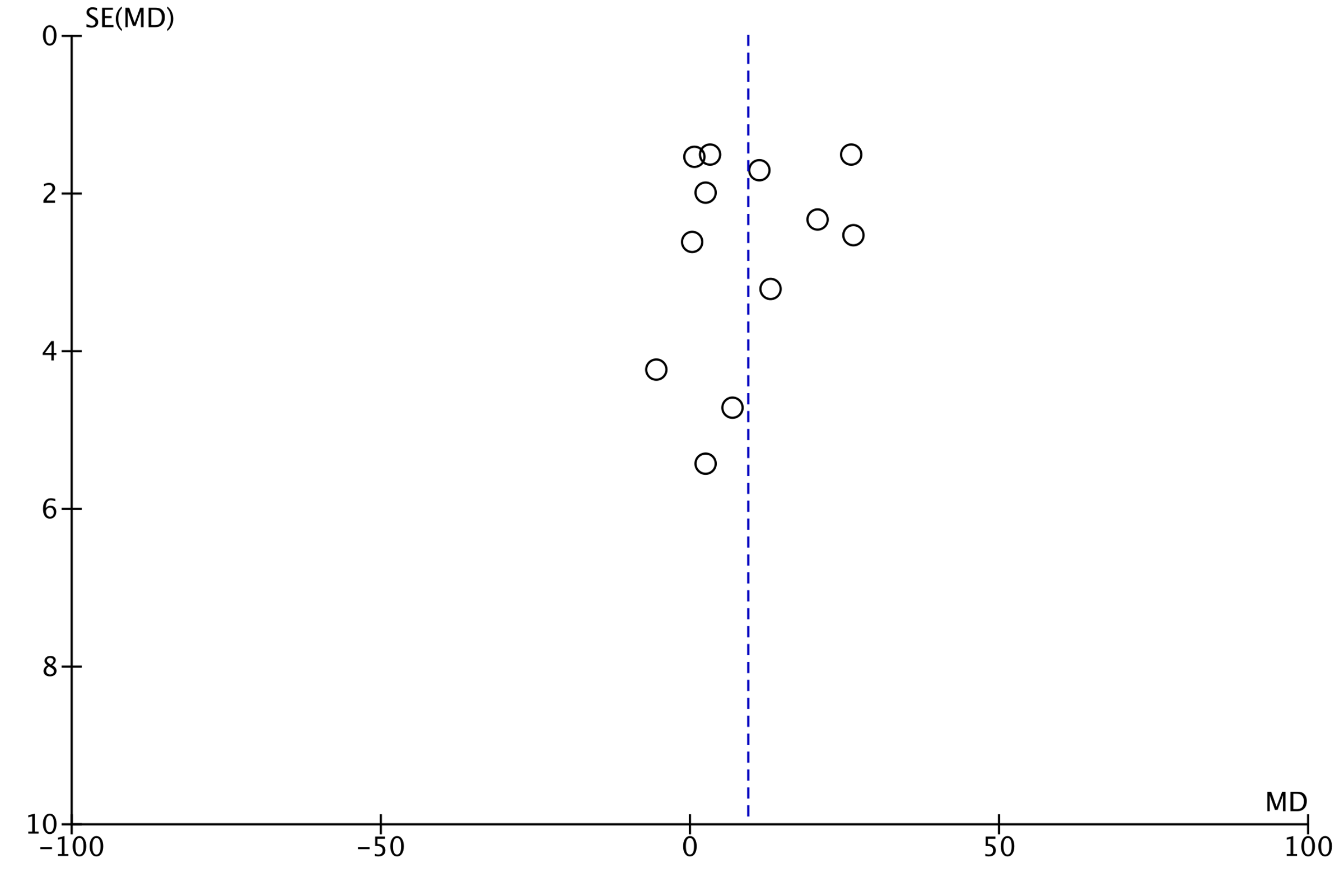


**Figure 31 Funnel plot of operative time**
